# Experimental and Computational Investigation of Sulfur Chemistry in the DART-MS Gas Stream: Implications for the Interpretation of the Mass Spectra of Organic Disulfides

**DOI:** 10.1021/jasms.6c00046

**Published:** 2026-04-14

**Authors:** Benedetta Garosi, Parandaman Arathala, Rabi A. Musah

**Affiliations:** Department of Chemistry, 5779 Louisiana State University, Baton Rouge, Louisiana 70803, United States

**Keywords:** DART-HRMS, disulfides, density functional theory, rate coefficient, variational transition state theory

## Abstract

Direct analysis in real time–high-resolution mass spectrometry (DART-HRMS) has proven useful for the detection and faithful representation of labile organosulfur compounds (OSCs). Nevertheless, it has been found that when exposed to the metastable helium (He*) of the DART gas stream under soft ionization conditions, some classes of OSCs such as disulfides, undergo several reactions to produce new organosulfur species, which complicate interpretation of their spectra. In this work, these new entities were characterized and the mechanisms of their formation explored. DART-HRMS analysis of diphenyl disulfide (PhSSPh) exhibited peaks consistent with [PhSSPh]^+•^, [PhSSPh + H]^+^, [PhSPh]^+•^, and [PhSOSPh + H]^+^ and various fragments and adducts including [PhSS]^+^ and [Ph_3_S_3_]^+^. *Ab initio*/DFT calculations coupled with variational transition state theory revealed that several of these peaks are artifacts of reactions occurring with He* and ^•^OH. Branching ratio analysis showed the dominant decomposition pathway of [PhSSPh]^+•^ to be [PhSSPh]^+•^ → PhS^•^ + PhS:, rather than [PhSSPh]^+•^ → PhS^+^=S + Ph^•^. The PhS^•^, Ph^•^, and ^•^OH formed serve as key intermediates in subsequent reactions that lead to the *m*/*z* values observed. From the results, a systematic approach for the interpretation of the DART mass spectra of organic disulfides was developed and successfully applied to various disulfide compound classes (e.g., aryl, alkyl, benzyl, and phenyl). Organic solvents were also observed to influence the ability to detect the compounds. Benzene; dichloromethane; hexane; and with some exceptions, ethyl acetate and tetrahydrofuran were found to be suitable, while disulfide signals in dimethylformamide were totally absent.

## Graphical abstract

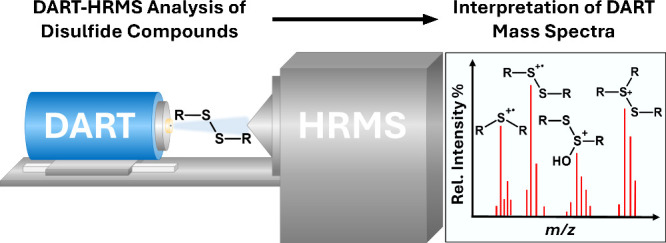

## 1 Introduction

Organic disulfides (R–S–S–R′) are a class of organosulfur compounds (OSCs) that play important roles in atmospheric, environmental, and biological chemistry. These molecules are of immense interest because of their contributions to climate change when emitted in the atmosphere;^1,2^ their involvement in protein folding, structure, and stability;^3^ their applications in drug delivery systems;^4,5^ and their utilization as food flavorants.^6^ The gas-phase chemistry of small-molecule OSCs in particular plays a significant role in atmospheric chemistry.^7,8^ OSCs are also widely used in chemical vapor deposition processes for the synthesis of semiconducting diamond films,^9^ and are employed in polymerization reactions as inhibitors, initiators, and chain-controlling agents.^10^ Because of their multidimensional roles and range of applications, there continues to be interest in understanding their mechanistic chemistry and the development of approaches for their monitoring and detection.

Experimental studies of OSCs are often hampered by the fact that they are highly labile, being converted even under mild atmospheric conditions into compounds that are artifacts of the analysis methods used to detect them. For this reason, experimental studies often benefit from computational calculations to help reveal mechanistic insights and provide complementary results, particularly in instances where the detection of products that would yield information on the nature of OSC transformations is challenging.^11–13^ For example, depending on the reaction conditions, the oxidation of disulfide molecules yields products such as thiosulfinates (R–S­(O)­S–R′), thiosulfonates (R–SO_2_S–R′), and sulfonic acids (R–SO_3_H).^14^ The intermediates formed during these processes are labile species that are difficult to detect using common analytical techniques. Analysis by gas chromatography – mass spectrometry (GC-MS) is well-known to result in their conversion into other molecules when the analytes are exposed to the temperatures in the GC inlet, causing misleading results and misinterpretations.^15^

A technique that has proven useful in detecting labile organosulfur species is direct analysis in real time – high-resolution mass spectrometry (DART-HRMS).^16,17^ DART is a soft ionization technique where ionization for the detection of analytes in positive-ion mode most commonly occurs by the Penning route, and/or protonation.^18^ In a DART experiment, helium gas is exposed to a plasma, resulting in the formation of metastable helium (He*), which then interacts with the analyte (M) to yield a cation radical (M^+•^). This Penning ionization can be summarized as:

$$\mathrm{He}+\mathrm{energy}\to {\mathrm{He}}^{*}$$ 1

$$M+{\mathrm{He}}^{*}\to M^{+•}+e^{-}+\mathrm{He}$$ 2

For ionization by protonation, metastable helium interacts with atmospheric water to form large, protonated water clusters which subsequently transfer a proton to the analyte to form [M + H]^+^ as illustrated below:

$${\mathrm{He}}^{*}+H_{2}O\to \mathrm{He}+H_{2}O^{+•}+e^{-}$$ 3

H2O+•+H2O→H3O++OH• 4

$$H_{3}O^{+}+nH_{2}O\to {\left[nH_{2}O+H\right]}^{+}$$ 5

$$M+{\left[nH_{2}O+H\right]}^{+}\to {\left[M+H\right]}^{+}+nH_{2}O$$ 6

Because routine DART-MS analyses are performed under soft ionization conditions and because the ionization mechanisms are well-understood, the DART mass spectrum for a given molecule can usually be readily predicted. For example, in positive ion mode, the DART mass spectrum of a pure compound would be anticipated to reveal one or two peaks, representative of M^+•^ and [M + H]^+^. For this reason, DART-HRMS is very useful in tracking reaction progress and assessing the purity of compounds. However, in the course of studies of purified OSCs, while DART mass spectra showed the presence of M^+•^ and [M + H]^+^, multiple additional unanticipated peaks were routinely observed. For example, in the analysis of various disulfides, the corresponding anticipated Penning ionization products and protonated precursors were detected, along with a number of other peaks with isotope distribution patterns indicative of the presence of more sulfur atoms than were contained within the original analytes. The presence of these entities complicates the interpretation of the spectra of disulfide compounds. While the observed high-resolution masses revealed their formulas, their structures were unknown, making it challenging to infer the mechanisms of their formation.

Quantum chemical calculations have proven to be a useful tool for the determination of the likely structures of gas phase entities that are detected in mass spectral analysis, and they can also offer insights into the kinetics of their formation.^11,19,20^ For example, in 1997 Frank et al.^21^ utilized *ab initio* and RRKM theory of chemical reactivity calculations to study the formation of [HO–S=O]^•^, [HO–S–OH]^•^, and [HSO_2_]^•^ generated in the gas phase by collisional reduction of the corresponding cations, coupled with the results of neutralization-reionization mass spectrometry (NRMS). Similarly, Zheng et al.^22^ investigated the pyrolysis of diethyl sulfide in a flow reactor by analyzing the product species (i.e., ethylene, methane, ethane, acetylene, carbon disulfide, thiophene, ethanethiol, methyl thiirane, ethyl methyl disulfide, and diethyl disulfide) by GC-MS and Fourier-transform infrared (FTIR) spectroscopy. Then, through thermodynamic and kinetic studies, they proposed the formation mechanisms of each species in order to model the reaction system.^22^ Jarvis et al.^23^ performed computational quantum calculations for the secondary products generated from the reactions of dimethyl disulfide with chemical background ions in a typical atmospheric pressure ionization (API)-MS experiment.^23^ In another relevant study, Cody and Fouquet^24^ reported an approach for the determination of the elemental composition of a compound (diphenyl ditelluride was used as an example) from its abundant isotope revealed by DART-HRMS analysis. However, to the best of our knowledge, there are no studies that offer a systematic interpretation of the results of organosulfur reactions taking place in the gas phase during DART-HRMS analysis.

We hypothesized that (1) new organosulfur species are generated when pure disulfides are exposed to He* in the DART gas stream; (2) high-level *ab initio*/DFT calculations can provide insights into the mechanism of their formation and facilitate determination of their structures; (3) tracer studies employing deuterated analogs can reveal gas phase adduct formation pathways; and (4) the entities formed may change as a function of the presence of various solvents. Accordingly, we analyzed a range of different types of OSCs by DART-HRMS and applied theoretical calculations to both the determination of the structures of the species detected, and the mechanisms of their formation. The results provide insights into the identities of the observed peaks and facilitate systematic interpretation of the DART mass spectra of disulfides.

## 2 Materials and Methods

### 2.1 Organosulfur Compounds

Di-*n*-butyl disulfide and diphenyl disulfide were acquired from AmBeed (Arlington Hts., IL). Dimethyl disulfide was purchased from Thermo Fisher Scientific Inc. (Waltham, MA). Di-*n*-propyl disulfide was obtained from Alfa Aesar (Ward Hill, MA). Di-*tert*-butyl disulfide was acquired from TCI America (Portland, OR). Diallyl disulfide was purchased from Cayman Chemical (Ann Arbor, MI). Di-*p*-tolyl disulfide was obtained from Sigma-Aldrich, Inc. (St. Louis, MO). Dimesityl disulfide (1,3,5-trimethyl isomer) was previously prepared following the procedure described by Block et al.^25^ Dibenzyl disulfide was previously synthesized as reported by Kubec et al.,^26^ and dibenzyl disulfide-*d*_14_ was prepared following the procedure described by He et al. 2018.^27^

### 2.2 DART-HRMS Mass Spectral Data Acquisition

High-resolution mass spectral data were acquired using a DART JumpShot ion source (IonSense, Saugus, MA) coupled with a JEOL AccuTOF high-resolution time-of-flight mass spectrometer (JEOL Ltd. USA, Peabody, MA). The data were obtained under soft ionization conditions at a DART ion source grid voltage of 250 V and collected in positive-ion mode, with the following mass spectrometer settings: ring lens, 5 V; orifice 1 voltage, 20 V; orifice 2 voltage, 5 V; peak voltage, 400 V; and detector voltage, 2000 V. All compounds were analyzed at DART gas temperatures between 150 and 350 °C (increasing in 25 °C increments) using ultrahigh purity helium gas (Airgas, Baton Rouge, LA) at a flow rate of 2 L/min. Mass spectral data were collected at a rate of 2 spectra per s over a mass range of *m*/*z* 40–1000. Polyethylene glycol (PEG 600; Thermo Fisher Scientific Inc., Waltham, MA) was used as the mass calibrant.

A capillary tube sampling technique was utilized to analyze the organosulfur compounds that were the subjects of this study: the closed end of a glass melting point capillary tube was inserted into the sample, and the coated surface was presented to the DART gas stream between the DART ion source and the mass spectrometer inlet. All sulfur-containing compounds were analyzed in the form in which they were received or synthesized. To confirm positive detection and identification of a given molecule and fragment, a 1% relative intensity and a threshold of ± 10 millimass units (mmu) were used.

### 2.3 Pure Disulfides in Organic Solvents

The behavior of organosulfur compounds in the DART He* gas stream was also studied for solubilized compounds. The solvents selected represent some of the most commonly used in organic synthesis and included benzene (Merck Millipore, Burlington, MA), dichloromethane, ethyl acetate, hexane (VWR Chemicals BDH, Radnor, PA), dimethylformamide, and tetrahydrofuran (Supelco, Burlington, MA). Using these solvents, solutions at a 1000 ppm concentration were prepared for each compound. The samples were analyzed by DART-HRMS as described above.

### 2.4 DART-HRMS Data Analysis

msAxel software (JEOL USA, Peabody, MA) was used for mass spectral data collection, calibration, averaging and background subtraction. The Mass Mountaineer software suite from RBC Software (Portsmouth, New Hampshire, USA) was used for data analysis and for generating mass spectral readouts.

### 2.5 Computational Methods

Computational calculations were carried out for diphenyl disulfide (DPDS) as a representative disulfide. All electronic structure calculations were performed using the Gaussian 16 program suite.^28^ The hybrid meta density functional (M06–2X)^29^ with the aug-cc-pV­(T+d)­Z basis set was used for geometry optimization, as well as vibrational analysis of molecules to explore the potential energy surfaces (PESs) of all the studied reaction pathways. M06–2X is an appropriate functional for thermochemical, kinetic, and noncovalent interaction calculations due to its robust performance across a broad range of chemical reactions.^30,31^ The aug-cc-pV­(T+d)­Z basis set includes additional tight *d*-functions, which are important for accurately capturing the electronic structure and bonding characteristics of sulfur-containing species.^8,32^ The single-point energy calculations for all the stationary points were performed at the CCSD­(T)/cc-pVTZ level^33^ on the optimized geometries determined at the M06–2X/aug-cc-pV­(T+d)­Z level. This was done to yield more accurate energetics information. The final energies for all the stationary points were calculated using single point energies to which a zero-point energy (ZPE) correction obtained at the M06–2X/aug-cc-pV­(T+d)­Z level was applied. All energies (along the PES profiles) that were associated with the primary dissociation of the DPDS cation radical were calculated at the CCSD­(T)/cc-pVTZ//M06–2X/aug-cc-pV­(T+d)­Z (denoted as CCSD­(T)//M06–2X) level. The energies of the secondary reaction path PES profiles were evaluated at the M06–2X/aug-cc-pV­(T+d)­Z level. Therefore, CCSD­(T)//M06–2X energies for the primary dissociation mechanism and M06–2X/aug-cc-pV­(T+d)­Z level energies for subsequent reactions were used throughout, unless otherwise stated.

### 2.6 Kinetics Determination Methodology

For reactions without an intrinsic barrier (barrierless reactions), variational transition state theory (VTST) was used to determine the PESs. Lengths of the reacting bonds were varied from 2.02 to 3.62 Å for S–S bonds, and from 1.76–4.16 Å for S–C bonds, at intervals of 0.01 Å for the primary dissociation of the DPDS cation radical. At each fixed S–S and S–C separation, all remaining internal coordinates were fully optimized. Subsequently, vibrational frequency calculations were performed to obtain the ZPE correction. The VTST approach was employed to calculate the barrierless reaction kinetics using the ktools/Multiwell package.^34^ According to VTST, the rate coefficient is minimized as a function of reaction coordinate(s) and temperature (T):

$$k^{\mathrm{VTST}}\left(T\right)=\min\;k^{\mathrm{TST}}\left(s,T\right)$$

where *k* ^VTST^ is the rate coefficient calculated using VTST and *k* ^TST^ is the rate coefficient calculated from transition state theory (TST). The inputs for the ktools/Multiwell are the PESs at the CCSD­(T)//M06–2X level and the vibrational frequencies obtained at the M06–2X/aug-cc-pV­(T+d)­Z level for the reactants and transition states. To compute VTST rate coefficients for barrierless reactions, ktools was employed by following the evolution of reactants toward a loosely bound transition-state region along the reaction coordinate. This procedure is implemented through a sequence of constrained geometry optimizations performed at selected points along the reaction path on the PES. For each constrained geometry, the corresponding potential energy was evaluated, rotational constants were derived from the optimized structure, and vibrational frequency calculations were carried out for all degrees of freedom orthogonal to the reaction coordinate after its removal. The VTST methodology has been described in detail elsewhere.^35^

## 3 Results and Discussion

### 3.1 DART-HRMS Analysis of Disulfide Compounds

The behavior of a variety of disulfide (RSSR) compounds was investigated upon exposure to the He* of the DART gas stream at different temperatures, and their mass spectra were interpreted. The represented classes of symmetrical disulfides studied are listed in Table 1, based on the identity of R, namely alkyl, such as methyl (−CH_3_), *n*-propyl (−(CH_2_)_2_CH_3_), *n*-butyl (**−**(CH_2_)_3_CH_3_), and *tert*-butyl (**−**C­(CH_3_)_3_); alkenyl, such as allyl (**−**CH_2_CH=CH_2_); aryl, such as phenyl (−C_6_H_5_); and alkyl aryl such as *p*-tolyl (−C_6_H_4_CH_3_), mesityl (−C_6_H_2_(CH_3_)_3_), benzyl (−CH_2_C_6_H_5_) and deuterated benzyl (−CD_2_C_6_D_5_). As a representative example, the DART – high-resolution mass spectrum of diphenyl disulfide is shown in Figure 1. The mass spectra for all the other disulfides are reported in Document A of the Supporting Information, and all of the corresponding mass data, including the *m*/*z* values and their relative intensities above a 1% threshold, are reported in Document B of the Supporting Information.

**Table 1:**
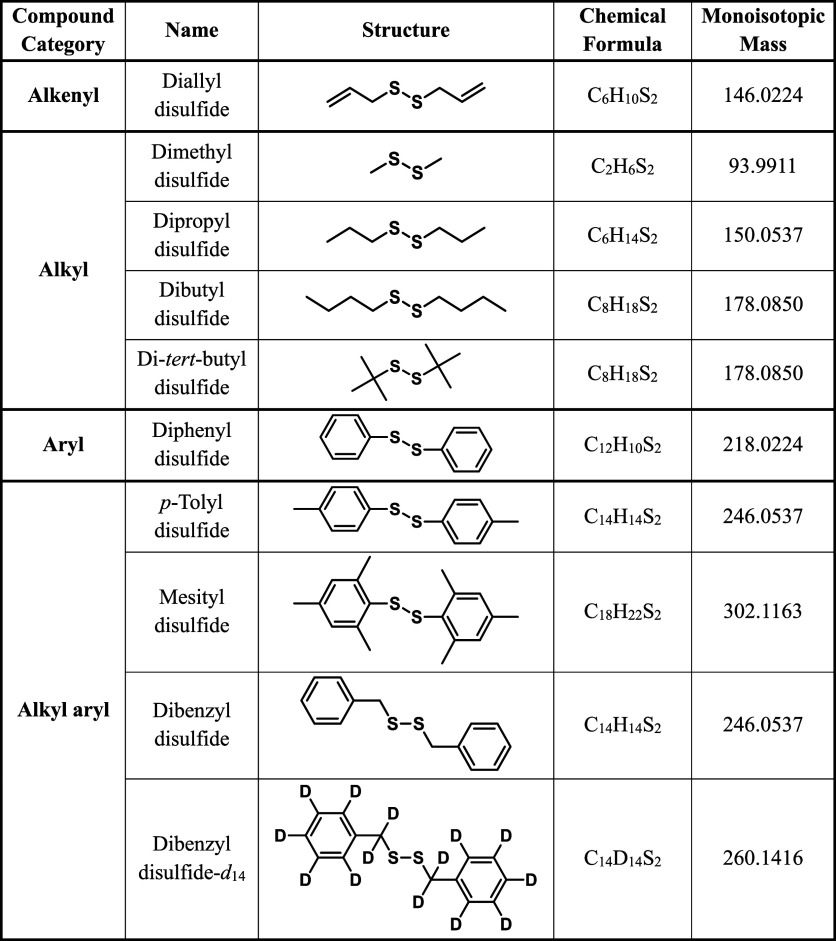
Disulfide Compounds that were the Subject of This Investigation.^a^

a The compound categories, molecule names, structures, chemical formulas and monoisotopic masses are reported.

**Figure 1:**
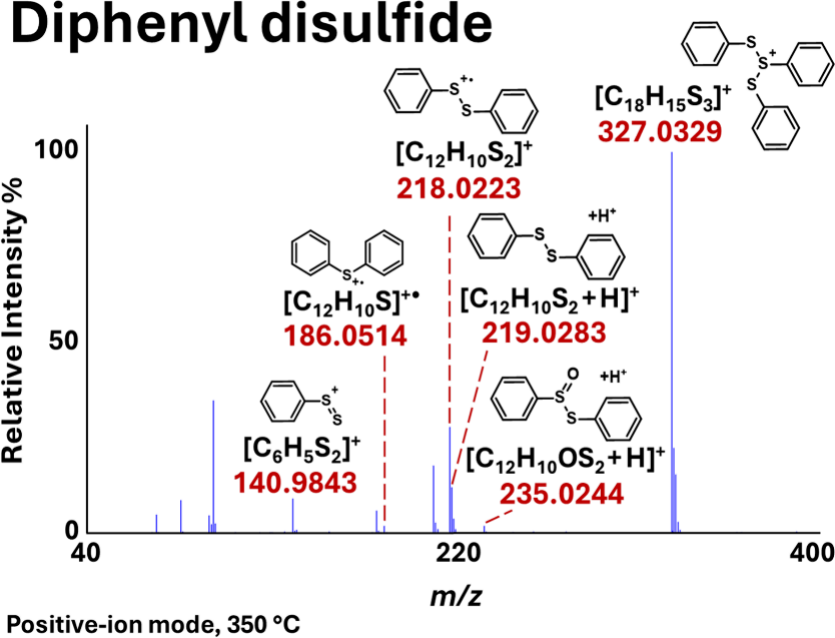
DART – high-resolution mass spectrum of diphenyl disulfide analyzed under soft ionization conditions in positive-ion mode at a He* gas temperature of 350 °C. Each identified peak is labeled with the respective observed *m*/*z* value, proposed formula, and proposed structure. The structures shown corresponding to the high-resolution masses were determined based on the results of the theoretical calculations (see below).

### 3.2 Interpretation of the Diphenyl Disulfide Mass Spectrum

Because pure diphenyl disulfide (PhSSPh; C_12_H_10_S_2_, *calc*. 218.0224) was analyzed by DART-HRMS in positive-ion mode under soft ionization conditions at 350 °C, it was anticipated that its mass spectrum would exhibit only one or two peaks representative of M^+•^ or [M + H]^+^. The high-resolution masses observed at *m*/*z* 218.0223 and *m*/*z* 219.0283 were consistent with the presence of these ions ([C_12_H_10_S_2_]^+•^ and [C_12_H_10_S_2_ + H]^+^, respectively), leading to their identification as the expected cation radical [PhSSPh]^+•^ and the protonated disulfide precursor [PhSSPh + H]^+^. However, the other detected peaks at *m*/*z* 140.9843, consistent with [C_6_H_5_S_2_]^+^; *m*/*z* 186.0514, corresponding to [C_12_H_10_S]^+•^; *m*/*z* 235.0244, consistent with [C_12_H_10_OS_2_ + H]^+^; and the base peak at *m*/*z* 327.0329, corresponding to [C_18_H_15_S_3_]^+^, were unanticipated and complicated the interpretation of the rest of the diphenyl disulfide spectrum. Thus, computational calculations were employed to confirm the identities and the structures corresponding to the monoisotopic masses detected by DART-HRMS.

### 3.3 Theoretical Calculations—Diphenyl Disulfide

At the temperature at which the DART-HRMS analysis was conducted (350 °C (623.15 K)), DPDS is susceptible to several possible bond-cleavage processes, including C–H, S–S, and S–C bond fissions. Three distinct unimolecular decomposition pathways were identified, as presented in Figure 2a. As illustrated, the reactions shown in eqs 7−9 indicate the S–S, S–C, and C–H bond dissociation options for the DPDS cation radical, leading to formation of their respective products. The bond dissociation energy (BDE) quantifies bond strength and serves as a key thermodynamic descriptor for predicting dominant decomposition and consumption pathways under high-temperature conditions. Therefore, the BDEs of S–S, S–C, and C–H bond scissions of the DPDS cation radical to form the corresponding products were calculated via eqs 7−9 in Figure 2a. The obtained values at the CCSD­(T)/cc-pVTZ//M06–2X/aug-cc-pV­(T+d)­Z level are provided on the PES shown in Figure 2b. The results indicate that the S–S, S–C, and C–H bond dissociation energies are 66.7, 76.2, and 120.0 kcal mol^–1^, respectively. These values suggest that S–S bond scission is the most feasible pathway due to its lower BDE compared to those of the S–C and C–H bonds. In contrast, C–H bond dissociation in the DPDS cation radical is highly endothermic and requires substantially higher energy (see Figure 2b). For comparison, the C–H bond dissociation energy in benzene has been reported to be ∼ 113.5 kcal mol^–1^.^36^ This indicates that the C–H bond dissociation of the DPDS cation radical is ∼6.5 kcal mol^–1^ higher in energy than the energy of C–H bond scission in benzene. Consequently, C–H bond dissociation in DPDS is expected to occur slowly, as C–H bonds possess significantly higher BDE’s than S–S and S–C bonds, making them much less susceptible to cleavage^37^ under typical reaction conditions (DART gas temperatures are between 150 and 350 °C). Therefore, the primary decomposition mechanism of the DPDS cation radical was investigated via both S–S and S–C bond scissions (see eqs 7 and 8 in Figure 2a), leading to the formation of their corresponding products, using high-level *ab initio*/DFT electronic structure calculations.

**Figure 2:**
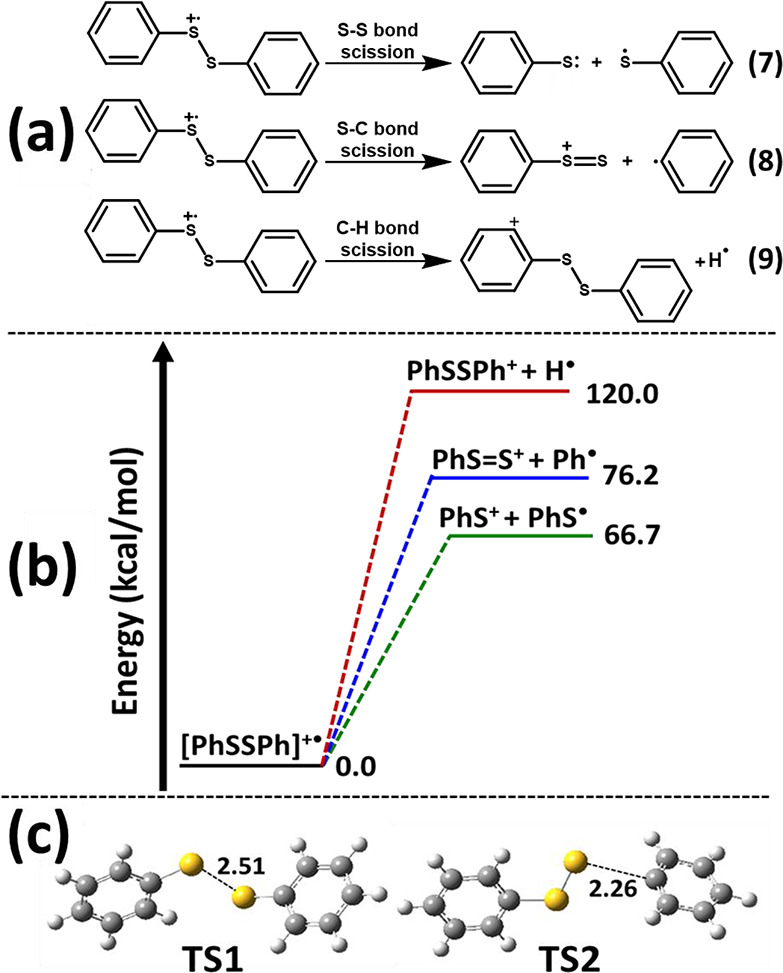
(a) Illustration of the possible primary dissociation pathways of the DPDS cation radical. (b) S–S, S–C, and C–H bond dissociation energies of the DPDS cation radical calculated at the CCSD­(T)/cc-pVTZ//M06–2X/aug-cc-pV­(T+d)­Z level. (c) One of the transition-state geometries for S–S and S–C bond scission of the DPDS cation radical optimized at the M06–2X/aug-cc-pV­(T+d)­Z level.

The primary dissociation of the DPDS cation radical proceeds through barrierless pathways during the S–S and S–C bond cleavage. The scanned PESs for the S–S and S–C bond cleavage reactions shown in eqs 7 and 8 are depicted in Figure 3. The bond lengths of 2.02–3.62 Å for the S–S and 1.76–4.16 Å for the S–C bonds along the reaction coordinate were scanned, and all transition states were located on the PES. These reaction pathways involve the formation of a cation radical transition state. An imaginary frequency was isolated for each transition state using the M06–2X/aug-cc-pV­(T+d)­Z method for vibrational analysis. Several transition states were identified for both the S–S and S–C bond dissociation pathways. For clarity, only one representative optimized transition state for each dissociation channel is shown (see Figure 2c).

**Figure 3:**
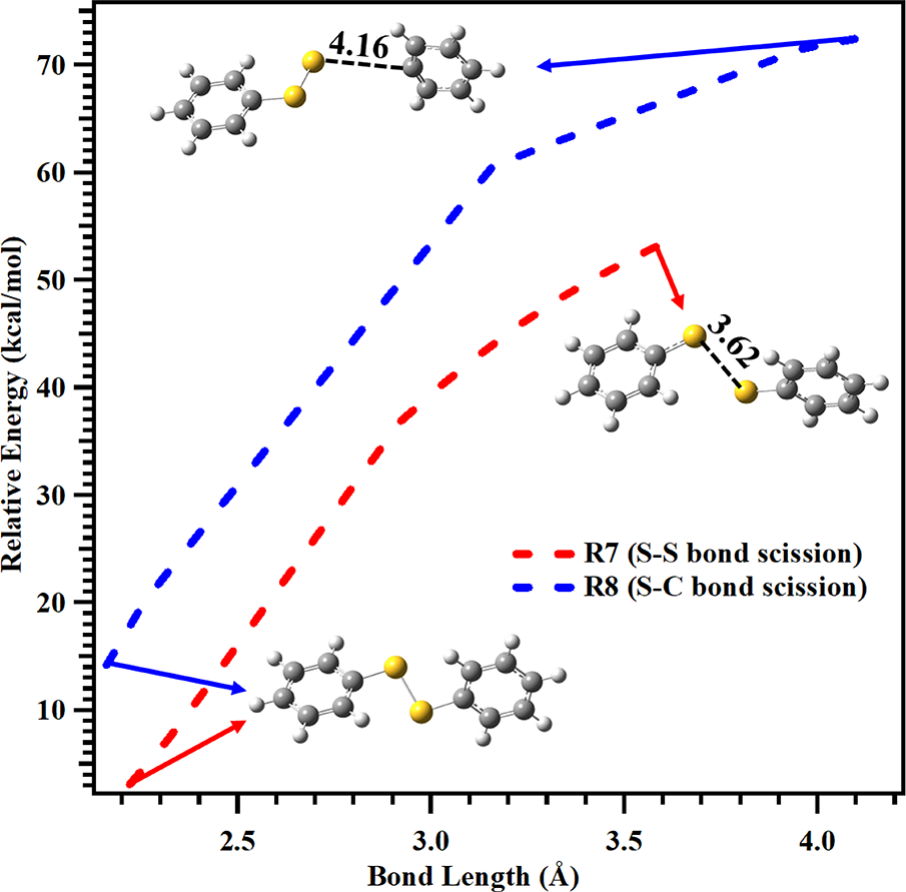
Scanned potential energy surface for the S–S and S–C bond dissociations of the DPDS cation radical leading to formation of their respective products, obtained at the CCSD­(T)/cc-pVTZ//M06–2X/aug-cc-pV­(T+d)­Z level. The relative energies of both reactions were calculated with respect to the energy of the diphenyl disulfide (DPDS) cation radical.

As illustrated in the figure, the S–S bond (TS1) and S–C bond (TS2) distances were constrained at 2.51 Å and 2.26 Å, respectively, while all remaining geometric parameters were fully optimized. The results indicate that the transition states associated with S–S bond cleavage are located at bond lengths ranging from 2.52–3.32 Å, with corresponding energies between 17.1 and 48.0 kcal mol^–1^. In contrast, for S–C bond cleavage, the transition states occur at bond lengths between 2.16 and 3.16 Å, with associated energies in the range of 20.2–60.6 kcal mol^–1^. The results from Figure 3 indicate that the energy required to break the S–S bond (see red line) is lower than that required to cleave the S–C bond in the DPDS cation radical at the CCSD­(T)//M06–2X level.

Rate coefficients k_R7_ and k_R8_ were calculated based on the scanned PESs for S–S and S–C bond dissociation reactions, respectively, using the VTST approach. The rate coefficients for these two reactions were calculated between 200 and 1500 K, and the corresponding values are displayed in Table 2.

**Table 2:** Rate Coefficients (in s^–1^) for the Unimolecular Dissociation of the DPDS Cation Radical via S–S (k_R7_), and S–C (k_R8_) Bond Cleavage to Form Their Respective Products in the 200–1500 K Temperature Range

T (K)	S–S bond cleavage, k_R7_ (s^–1^)	S–C bond cleavage, k_R8_ (s^–1^)
200	2.20 × 10^–34^	3.73 × 10^–42^
298	9.66 × 10^–19^	6.92 × 10^–24^
300	1.58 × 10^–18^	1.22 × 10^–23^
400	1.42 × 10^–10^	2.54 × 10^–14^
500	8.63 × 10^–6^	1.05 × 10^–8^
600	1.35 × 10^–2^	6.14 × 10^–5^
625	5.88 × 10^–2^	3.49 × 10^–4^
700	2.59 × 10 °	3.06 × 10^–2^
800	1.34 × 10^2^	3.28 × 10 °
900	2.90 × 10^3^	1.25 × 10^2^
1000	3.39 × 10^4^	2.33 × 10^3^
1100	2.52 × 10^5^	2.53 × 10^4^
1200	1.30 × 10^6^	1.79 × 10^5^
1300	4.86 × 10^6^	8.71 × 10^5^
1400	1.34 × 10^7^	2.97 × 10^6^
1500	2.81 × 10^7^	7.43 × 10^6^

According to the Table, rate coefficients for the S–S and S–C bond scission reactions exhibited a positive temperature dependence, meaning that the rate coefficients increased with increasing temperature. The rate coefficients for S–S bond cleavage were found to be ∼1–7 orders of magnitude larger compared to the rate coefficient values for the S–C bond cleavage reaction in the same studied temperature range. Specifically, at 625 K, which is relevant to the DART gas stream temperature at which DPDS was analyzed, the rate coefficients for the S–S and S–C bond dissociation of the DPDS cation radical to form their respective products, were found to be 5.9 × 10^–2^ and 3.5 × 10^–4^ s^–1^ respectively. The larger rate coefficients for S–S bond cleavage are due to the lower barrier heights compared to the values of the S–C bond cleavage reaction barriers (see Figure 3). The branching ratios indicate that S–S and S–C bond fission reactions occur at 99 and 0.006%, respectively at 625 K. This suggests that the products formed from the S–S and S–C bond cleavage reactions are responsible for the formation of the additional mass spectral features that appear in the analysis of pure DPDS by DART-HRMS (see Figure 1) (e.g., *m*/*z* 186.0514, *m*/*z* 235.0244, and *m*/*z* 327.0329). In particular, the results indicate that S–S and S–C bond cleavage of the DPDS cation radical in the DART gas stream can generate reactive intermediates such as PhS^+^, PhS^•^, PhS=S^+^, and Ph^•^ (see Figure 2) which serve as the primary precursors that engage in further reactions that lead to the additional ions observed in the mass spectrum.

While it is generally accepted that elevated pressure and temperature can promote bond scission for certain types of compounds, we believe that for the work reported here, bond scission is promoted by the metastable helium. In fact, not only were the DART-HRMS experiments carried out at ambient pressure, but the fragments detected during our analyses also proved to be independent of temperature, as identical fragmentation patterns were observed when the disulfides were analyzed at DART gas temperatures between 150 and 350 °C (see mass values obtained at each temperature in Document B of the Supporting Information).

Thus, with the rate coefficient results in hand, we initiated an evaluation of the energies associated with formation of the diphenyl sulfide (DPS) cation radical ([PhSPh]^+•^, consistent with *m*/*z* 186.0514) from the combination of the phenyl thiyl cation [PhS]^+^ and the phenyl radical [Ph]^•^ intermediates. The spontaneous nature of this reaction was rationalized based on the calculated energies. The formation of the DPS cation radical from these reactive intermediates is depicted in Scheme 1, and the corresponding scanned PES computed at the M06–2X/aug-cc-pV­(T+d)­Z level, is presented in Figure 4. As illustrated, addition of PhS^+^ to the C-centered Ph radical leading to DPS cation radical formation was found to be a barrierless process. The C–S bond length was scanned from 1.73 to 4.33 Å along the reaction coordinate (see Figure 4), and all initial transition-states were located on the PES. The transition states for this reaction along with other possible channels studied in this work were optimized at the M06–2X/aug-cc-pV­(T+d)­Z level and are shown in Figure S1. The results indicate that the transition states associated with C–S bond formation occur at bond lengths ranging from 2.33 to 3.33 Å, with corresponding transition state energies between −125.2 and −85.0 kcal mol^–1^. This indicates that this reaction is dominant under the present experimental conditions.

**Scheme 1:**
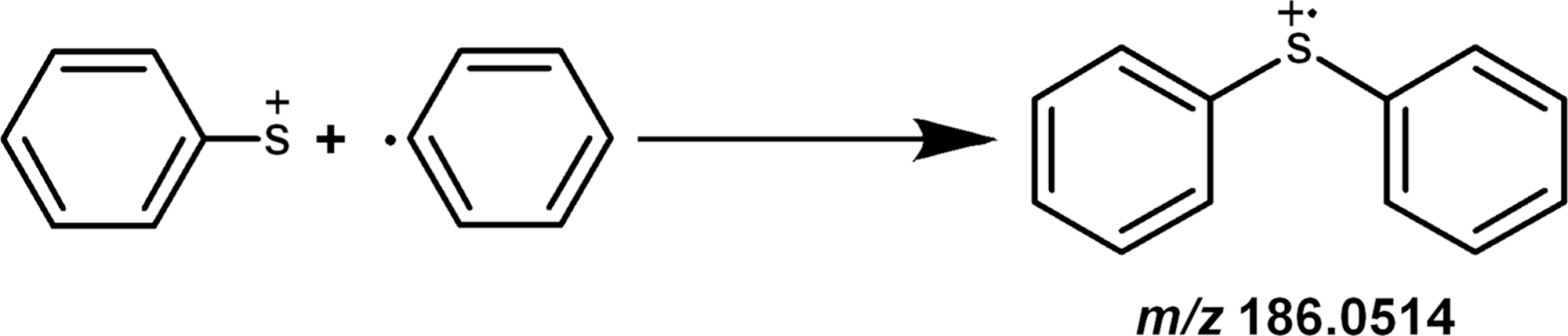
Formation of the Diphenyl Sulfide (DPS) Cation Radical from the Reactive Intermediates Phenyl Thiyl Cation (PhS^+^) and Phenyl Radical (Ph^•^)

**Figure 4:**
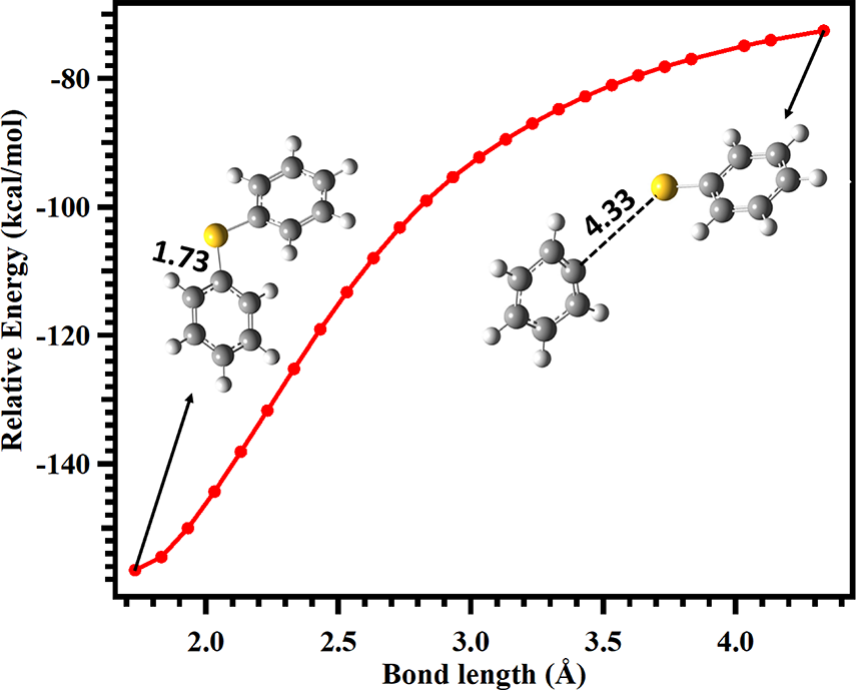
Scanned potential energy surface for phenyl thiyl cation (PhS^+^) addition to the C atom of the phenyl radical (Ph^•^) at the M06–2X/aug-cc-pV­(T+d)­Z level of theory. The relative energy of each point was calculated with respect to the PhS^+^ + Ph^•^ reactants.

We next investigated the formation mechanism for the ion represented by *m*/*z* 235.0244, corresponding to [C_12_H_10_OS_2_ + H]^+^. This product may be generated from the reaction of the DPDS cation radical with OH radical in the DART gas stream as depicted in Scheme 2. The PES profile for this reaction was surveyed at the M06–2X/aug-cc-pV­(T+d)­Z level and is shown in Figure S2. The S–O bond length was scanned from 1.61 to 2.91 Å along the reaction coordinate (see Figure S2), and all initial transition states were located on the PES. The results indicate that the transition states associated with S–O bond formation occur at bond lengths ranging from 2.11 to 2.61 Å, with corresponding transition state energies between −7.0 and −26.1 kcal mol^–1^. The results suggest that the formation of PhS^+^(OH)­SPh from the [PhSSPh]^+•^ + ^•^OH reaction, reported in Scheme 2, is feasible under the present experimental conditions.

**Scheme 2:**
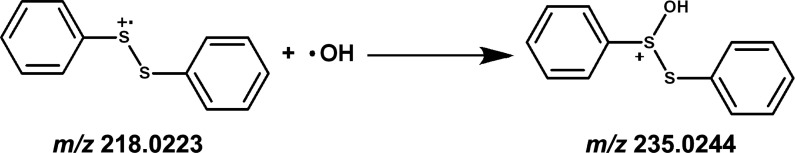
Formation of PhS^+^(OH)­SPh from the Reactive Intermediates Diphenyl Disulfide Cation Radical [PhSSPh]^+•^ and Hydroxyl Radical (^•^OH).

Under soft energy ionizing conditions, DPDS undergoes S–S and S–C bond scission to generate their respective reactive intermediates. These species are extremely short-lived and they exhibit high reactivity. For this reason, covalent bond-formation reactions can still occur in the DART gas stream environment because the increased kinetic energy of the particles leads to more frequent and energetic collisions, thereby enhancing the likelihood of productive interactions and increasing the overall reaction rate. The formation of products from these intermediates is governed by thermodynamic stability and the associated activation barriers. In this context, the calculated transition state energies for S–O bond formation range from −7 to −26.0 kcal mol^–1^, indicating an essentially barrier-free process that facilitates spontaneous product formation.

While *m*/*z* 235.0244 is presented in Figure S2 as containing a S–OH moiety, it is also possible that its structure is best described as [PhS­(=O)­SPh + H]^+^. To assess this possibility, a PES scan was performed by systematically elongating the O–H bond from 0.97 to 2.37 Å along the reaction coordinate. The resulting PES profile indicates that O–H bond scission in PhS^+^(OH)­SPh leads to the formation of a PhSH••(O=S)­Ph adduct. This transformation occurs through initial O–H bond fission, followed by intramolecular hydrogen-atom transfer to the adjacent sulfur atom and subsequent S–S bond cleavage (see Figure S3). All initially proposed transition state structures were successfully optimized and confirmed on the PES. The transition states associated with O–H bond cleavage are located at O–H bond lengths between 1.37 and 1.47 Å, with corresponding activation energies in the range of 36.5–49.1 kcal mol^–1^ relative to the energy of the PhS^+^(OH)­SPh reactant. These results clearly suggest that the structure of *m*/*z* 235.0244 represents an equilibrium between the PhS^+^(OH)­SPh and PhSH••(O=S)­Ph adducts (see Figure S3).

For the peak at *m*/*z* 327.0329, we assigned Ph–S^+^(S–Ph)–S–Ph as a tentative structure. The most probable pathway for the formation of this product involves the addition of a phenyl thiyl radical to the sulfur atom of the DPDS cation radical, leading to the formation of a new S–S single bond, as illustrated in Scheme 3. We investigated the energetics of this reaction, and the corresponding scanned PES profile computed at the M06–2X/aug-cc-pV­(T+d)­Z level is shown in Figure S4. As illustrated in the figure, two possible reaction pathways originate from the PhSSPh^+•^ + PhS^•^ reactants. In the first, the reaction proceeds barrierlessly to form the Ph–S^+^(S–Ph)–S–Ph product (see Figure S4). In the second, PhSSPh^+•^ and PhS^•^ associate to form an adduct, PhSSPh^+•^••PhS^•^. To characterize the S–S bond formation, the S–S bond distance was scanned from 2.10 to 3.50 Å along the reaction coordinate (Figure S4), and all transition-states were successfully located on the PES. The results indicate that the transition states associated with S–S bond formation occur at bond lengths between 2.90 and 3.20 Å, with corresponding energies ranging from −11.5 to −16.4 kcal mol^–1^ relative to the energy of the separated PhSSPh^+•^ + PhS^•^ reactants. In contrast, the formation of the PhSSPh^+•^••PhS^•^ adduct is energetically less favorable than direct formation of the Ph–S^+^(S–Ph)–S–Ph product (see Figure S4). Therefore, the barrierless formation of the Ph–S^+^(S–Ph)–S–Ph product represents the dominant reaction pathway under the present experimental conditions.

**Scheme 3:**
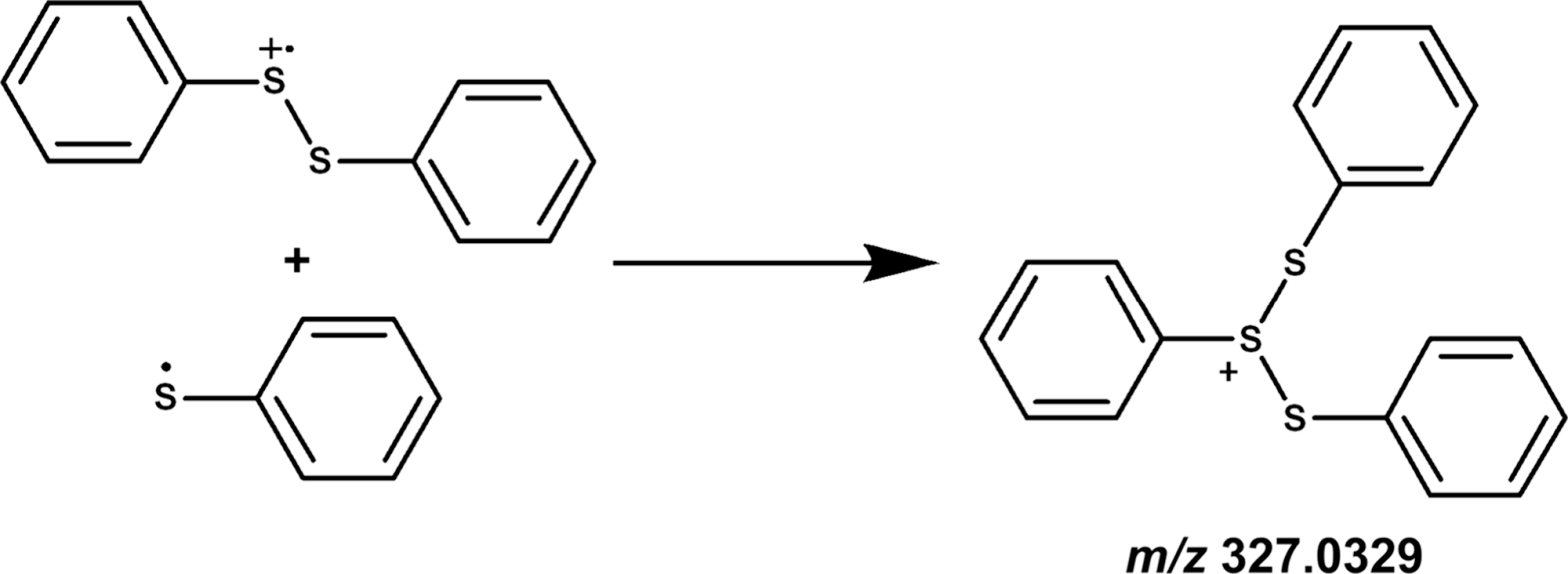
Formation of Ph–S^+^(S–Ph)–S–Ph from the Reactive Intermediates Diphenyl Disulfide Cation Radical [PhSSPh]^+•^ and Phenyl Thiyl Radical PhS^•^

### 3.4 Tracer Studies with Dibenzyl Disulfide and Dibenzyl Disulfide-*d*_14_

To experimentally investigate the recombination of ion fragments and radical intermediates following their formation in the DART gas stream, deuterated counterparts of model compounds were employed. Accordingly, tracer studies using dibenzyl disulfide-*d*_14_ (Bn_*d7*_SSBn_*d7*_; C_14_D_14_S_2_, *calc*. 260.1416) were conducted. The DART mass spectrum of dibenzyl disulfide-*d*_14_ (see Figure S5) exhibited peaks analogous to those formed for its nondeuterated analog (see Figure S6) including peaks consistent with the presence of the unfragmented protonated and cation radical precursors, and various product ions formed from their subsequent fragmentation. Therefore, it was anticipated that analysis of a mixture of the deuterated and nondeuterated compounds would reveal peaks consistent with organosulfur entities derived not only from both the unlabeled dibenzyl disulfide and its deuterated counterpart dibenzyl disulfide-*d*_14_, but also peaks for entities formed from recombinations of deuterated and nondeuterated fragments.

Figure 5 shows a head-to-tail plot that features the results of DART-HRMS analysis of a 1:1 mixture of dibenzyl disulfide and dibenzyl disulfide-*d*_14_. The top spectrum in blue is that of the mixture, while on the bottom are the overlaid spectra of BnSSBn in red and Bn_*d*_SSBn_*d*_ in black. The mass data information for the numbered peaks (1 through 31) in the top spectrum in Figure 5 is listed in Table 3, including experimentally observed *m*/*z* values, the corresponding molecular formulas, and the proposed chemical structures. Peaks identified in the spectrum of the dibenzyl mixture that originated from dibenzyl disulfide only are *m*/*z* 91.0548, consistent with the tropylium cation radical [C_7_H_7_]^+^; *m*/*z* 181.1021, corresponding to [PhCH=CHPh + H]^+^; *m*/*z* 213.0740, consistent with deprotonated dibenzyl sulfide [BnS=CHPh]^+^; *m*/*z* 245.0461, 246.0535, and 247.0614 that correspond to deprotonated dibenzyl disulfide [BnSS=CHPh]^+^, the dibenzyl disulfide cation radical [BnSSBn]^+•^, and protonated dibenzyl disulfide [BnSSBn + H]^+^, respectively; *m*/*z* 264.0881, corresponding to ammoniated dibenzyl disulfide [BnSSBn + NH_4_]^+^; *m*/*z* 337.1087, with formula [Bn_2_SSBn]^+^ consistent with a dibenzyl disulfide molecule bonded to a benzyl group through the sulfur atom; and *m*/*z* 369.0809, which corresponds to [Bn_3_S_3_]^+^ and represents dibenzyl disulfide plus a benzyl thiolate group.

**Figure 5:**
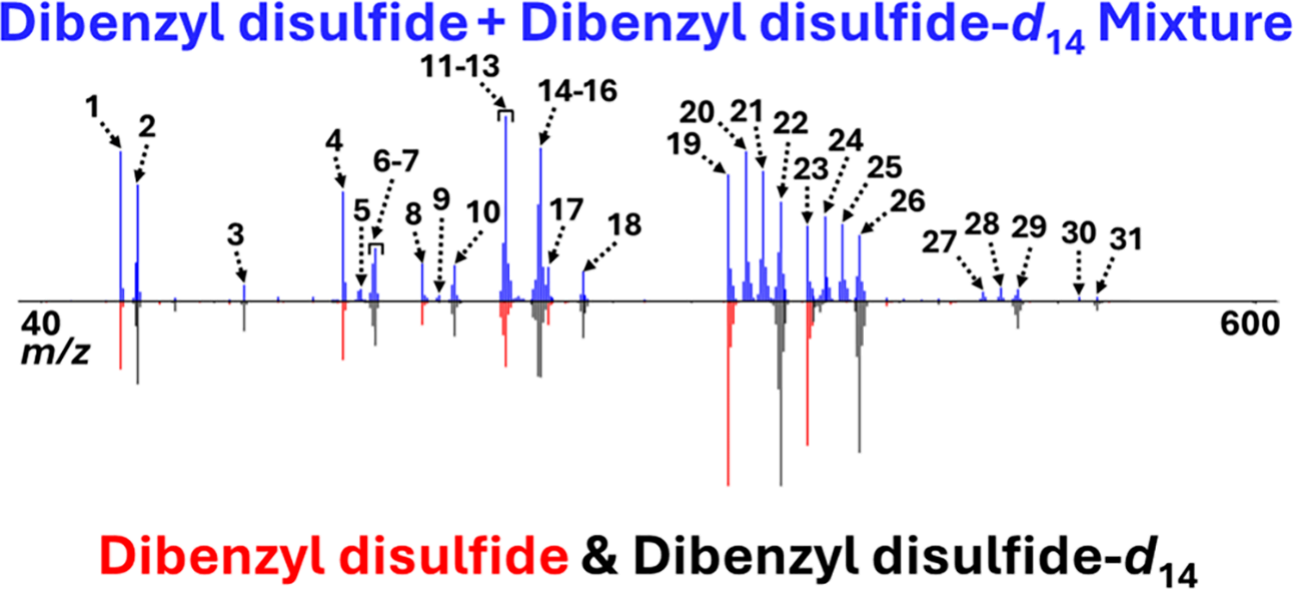
Head-to-tail plot illustrating DART – high-resolution mass spectra of the dibenzyl disulfide – dibenzyl disulfide-*d*_14_ mixture (top spectrum in blue) versus that of dibenzyl disulfide (bottom in red), overlaid with the mass spectrum of dibenzyl disulfide-*d*_14_ (bottom in black). The assigned peak numbers refer to the peak identities reported in Table 3.

**Table 3:**
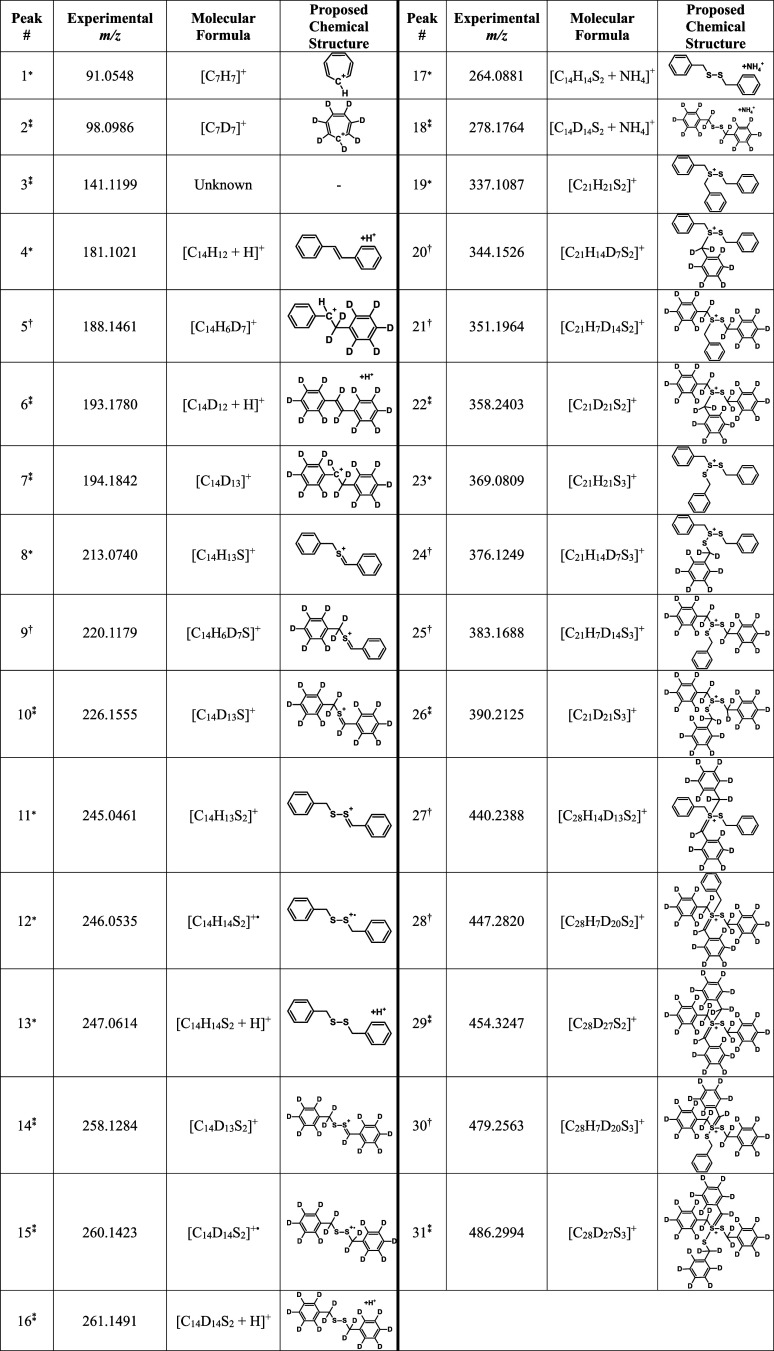
Mass Spectral Data of the Peaks Revealed upon DART-HRMS Analysis of Dibenzyl Disulfide + Dibenzyl Disulfide-*d*_14_ ^a^

a *Indicates a peak derived solely from the DART-HRMS analysis of the dibenzyl disulfide molecule present in the mixture. _*_^*^Indicates a peak derived solely from the DART-HRMS analysis of the unlabeled dibenzyl disulfide-*d*_14_ molecule present in the mixture. †Indicates a peak formed in the DART gas stream due to the reactions and combinations of fragments originating from both the dibenzyl disulfide and dibenzyl disulfide-*d*_14_ molecules in the mixture.

Peaks observed in the DART-HRMS analysis of the BnSSBn/Bn_*d7*_SSBn_*d7*_ mixture that are due to the presence of dibenzyl disulfide-*d*_14_ include: *m*/*z* 98.0986, consistent with the deuterated tropylium cation radical [C_7_D_7_]^+^; *m*/*z* 193.1780, corresponding to [Ph_*d5*_CD=CDPh_*d5*_ + H]^+^; *m*/*z* 194.1842, corresponding to [Ph_*d5*_CD–CD_2_Ph_*d5*_]^+^; *m*/*z* 226.1555, consistent with deprotonated dibenzyl sulfide-*d*_13_ [Bn_*d7*_S=CDPh_*d5*_]^+^; *m*/*z* 258.1284, 260.1423, and 261.1491 that correspond to deprotonated dibenzyl disulfide-*d*_13_ [Bn_*d7*_SS=CDPh_*d5*_]^+^, the benzyl disulfide-*d*_14_ cation radical [Bn_*d*_SSBn_*d*_]^+•^, and protonated dibenzyl disulfide-*d*_14_ [Bn_*d7*_SSBn_*d7*_ + H]^+^, respectively; *m*/*z* 278.1764, corresponding to ammoniated dibenzyl disulfide-*d*_14_ [Bnd7SSBnd7 + NH_4_]^+^; *m*/*z* 358.2403, with structure [Bn_*d7*_S­(–Bn_*d7*_)–SBn_*d7*_]^+^ corresponding to dibenzyl disulfide-*d*_14_ bonded to one deuterated benzyl group through the sulfur atom; *m*/*z* 390.2125 consistent with [Bn_*d7*_S­(–SBn_*d7*_)–SBn_*d7*_]^+^ which is a dibenzyl disulfide-*d*_14_ bonded to a deuterated benzyl thiolate group; *m*/*z* 454.3247, with formula [(Bn_*d7*_)_2_S­(=CDPh_*d5*_)–SBn_*d7*_]^+^ corresponding to dibenzyl disulfide-*d*_14_ plus two deuterated benzyl groups; and *m*/*z* 486.2994, [Bn_*d7*_(Bn_*d7*_S)–S­(=CDPh_*d5*_)–SBn_*d7*_]^+^ consistent with dibenzyl disulfide-*d*_14_ plus a deuterated benzyl thiolate group and a deuterated benzyl group.

The remaining peaks observed in the mass spectrum of the organosulfur compound mixture are consistent with masses of new organosulfur species resulting from combinations of fragments generated from BnSSBn and Bn_*d7*_SSBnd in the DART He* gas stream. These entities are *m*/*z* 188.1461, corresponding to [PhCH–CD_2_Ph_*d5*_]^+^; *m*/*z* 220.116, consistent with [Bn_*d7*_S=CHPh]^+^; *m*/*z* 344.1526, corresponding to an adduct composed of dibenzyl disulfide plus a deuterated benzyl group [BnS­(–Bn_*d7*_)–SBn]^+^; *m*/*z* 351.1964, consistent with dibenzyl disulfide-*d*_14_ plus a nondeuterated benzyl group [Bn_*d7*_S­(–Bn)–SBn_*d7*_]^+^; *m*/*z* 376.1249, corresponding to a molecule composed of dibenzyl disulfide plus a deuterated benzyl thiolate group [BnS­(–SBn_*d7*_)–SBn]^+^; *m*/*z* 383.1688, consistent with an adduct composed of deuterated dibenzyl disulfide plus a nondeuterated benzyl thiolate group [Bn_*d7*_S­(–SBn)–SBn_*d7*_]^+^; *m*/*z* 440.2388, corresponding to a molecule composed of dibenzyl disulfide plus two deuterated benzyl groups [Bn­(Bn_*d7*_)–S­(=CDPh_*d5*_)–SBn]^+^; *m*/*z* 447.2820, consistent with an adduct composed of dibenzyl disulfide-*d*_14_ plus one deuterated and one nondeuterated benzyl group [Bn_*d7*_(Bn)–S­(=CDPh_*d5*_)–SBn_*d7*_]^+^; and *m*/*z* 479.2563, consistent with a molecule composed of dibenzyl disulfide-*d*_14_ plus a deuterated benzyl group and a nondeuterated benzyl thiolate group [Bn_*d7*_(BnS)–S­(=CDPh_*d5*_)–SBn_*d7*_]^+^.

### 3.5 Systematic Interpretation of DART – High-Resolution Mass Spectra of Other Pure Disulfides

The computational calculations and tracer studies with the nondeuterated and deuterated compounds implied that in the DART gas stream, disulfides undergo a series of reactions beginning with protonation and Penning ionization, resulting in a predictable sequence of steps that yield anticipated adducts of various kinds that are responsible for their relatively complex mass spectra. As the cation radical [R–S–S–R]^+•^ is generated through the Penning route, it undergoes fragmentation via S–S and S–C bond scissions, or it reacts with ^•^OH present in the DART gas stream resulting in an oxygenated species, [R–S­(OH)–S–R]^+^. The fragments formed, such as R–S^+•^, ^•^S–R, R–(S^+^)=S, and ^•^R then interact with the starting material to yield a combination of molecules such as [R_2_S_3_ + H]^+^, [R_3_S_2_]^+^, and [R_3_S_3_]^+^. The understanding of these steps can in principle be utilized for the interpretation of the DART mass spectra of disulfides in general. Therefore, to determine whether the theoretical and tracer study findings are broadly applicable to disulfides, a variety of disulfide OSC structures were surveyed to determine whether their spectra could be predicted and readily interpreted.

Accordingly, Figure 6 shows the DART – high-resolution mass spectra of dimethyl disulfide (Panel A), di-*n*-propyl disulfide (Panel B), di-*tert*-butyl disulfide (Panel C), and dimesityl disulfide (Panel D). Their mass spectra showed the presence of ions reflective of the occurrence of ionization through the typical means (i.e., M^+•^ via Penning ionization and [M + H]^+^ via protonation), as well as ions formed from the same pathways revealed through the aforementioned tracer and computational studies of dibenzyl disulfide. The structures of these entities and their corresponding observed high-resolution masses are shown in Figure 6. Analogous results were obtained for the DART – high-resolution mass spectra of dibenzyl disulfide-*d*_14_, dibenzyl disulfide, diallyl disulfide, di-*n*-butyl disulfide, and di-*p*-tolyl disulfide (see Figures S5–S9 respectively). The mass data for all of the mass spectra collected at DART gas temperatures between 150 and 350 °C, including the *m*/*z* values and their relative intensities above a 1% threshold, are reported in Document B of the Supporting Information. The results reveal a logical and systematic approach for interpreting the mass spectra and making peak assignments.

**Figure 6:**
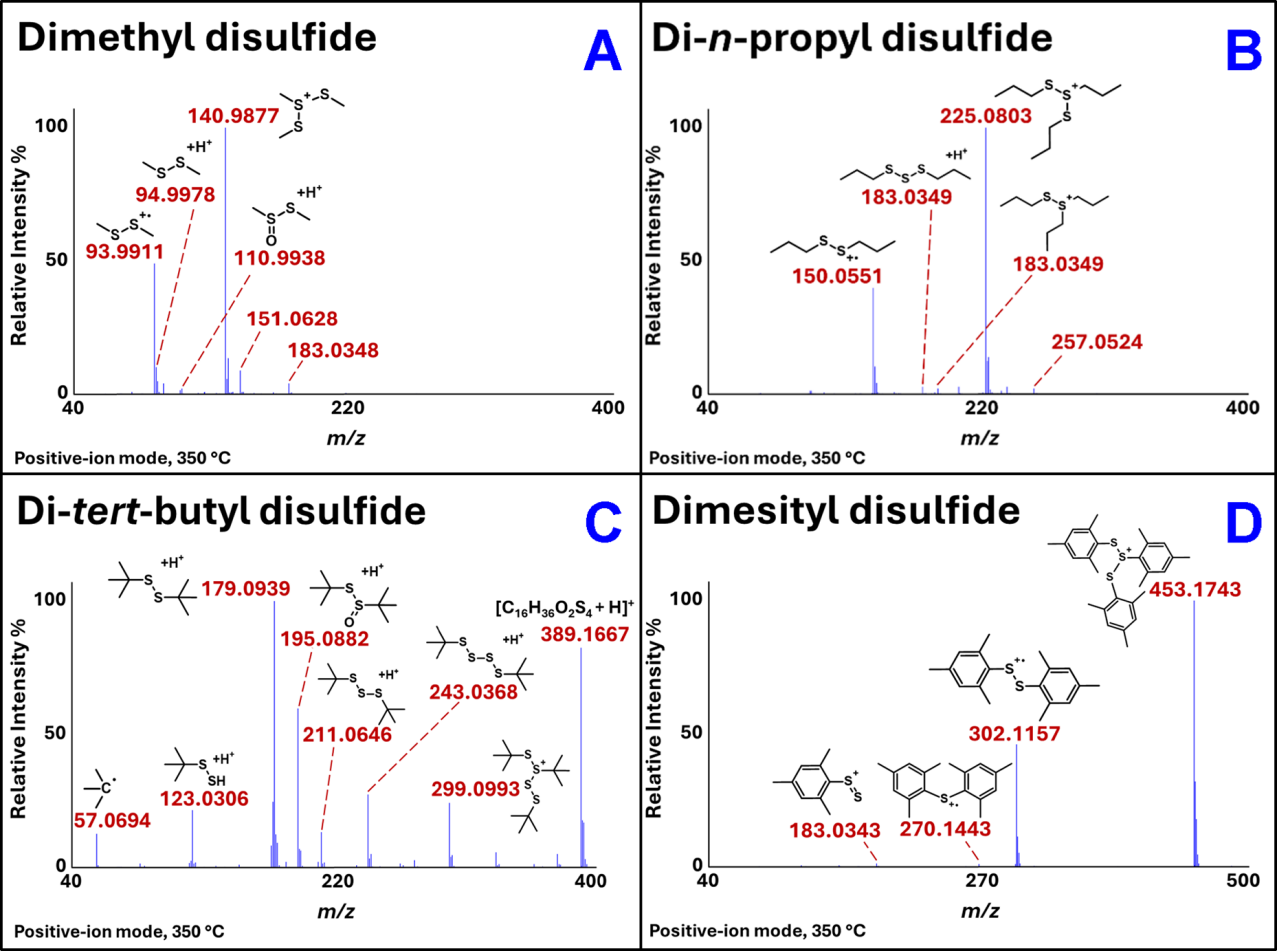
DART mass spectra of dimethyl disulfide (Panel A), di-*n*-propyl disulfide (Panel B), di-*tert*-butyl disulfide (Panel C), and dimesityl disulfide (Panel D). The corresponding mass data tables appear in Document B of the Supporting Information.

### 3.6 Interpretation of the DART – Mass Spectra of Disulfides Solubilized in Organic Solvents

When DART-HRMS is used to monitor reaction progress, the samples analyzed are often solubilized. Therefore, it was of interest to determine whether the behavior of disulfides dissolved in common organic solvents was similar to that described above for neat compounds. The solvents selected are some of the most commonly used in organic synthesis including benzene (C_6_H_6_, *calc*. 78.0470), dichloromethane (DCM; CH_2_Cl_2_, *calc*. 83.9534), dimethylformamide (DMF; C_2_H_7_NO, *calc*. 73.0528), ethyl acetate (C_4_H_8_O_2_, *calc*. 88.0524), hexane (C_6_H_14_, *calc*. 86.1096), and tetrahydrofuran (THF; C_4_H_8_O, *calc*. 72.0575). A concentration of each disulfide of 1000 ppm was analyzed by DART-HRMS, and the mass spectra obtained were compared to those of the respective neat disulfide standards. Figure 7 shows representative mass spectra for diphenyl disulfide solutions series, while the data for all the other organosulfur compounds are reported in Figures S10 to S17. The mass data for all the compounds analyzed in the full range of organic solvents, including the *m*/*z* values and their relative intensities above a 1% threshold, are reported in Document C of the Supporting Information.

**Figure 7:**
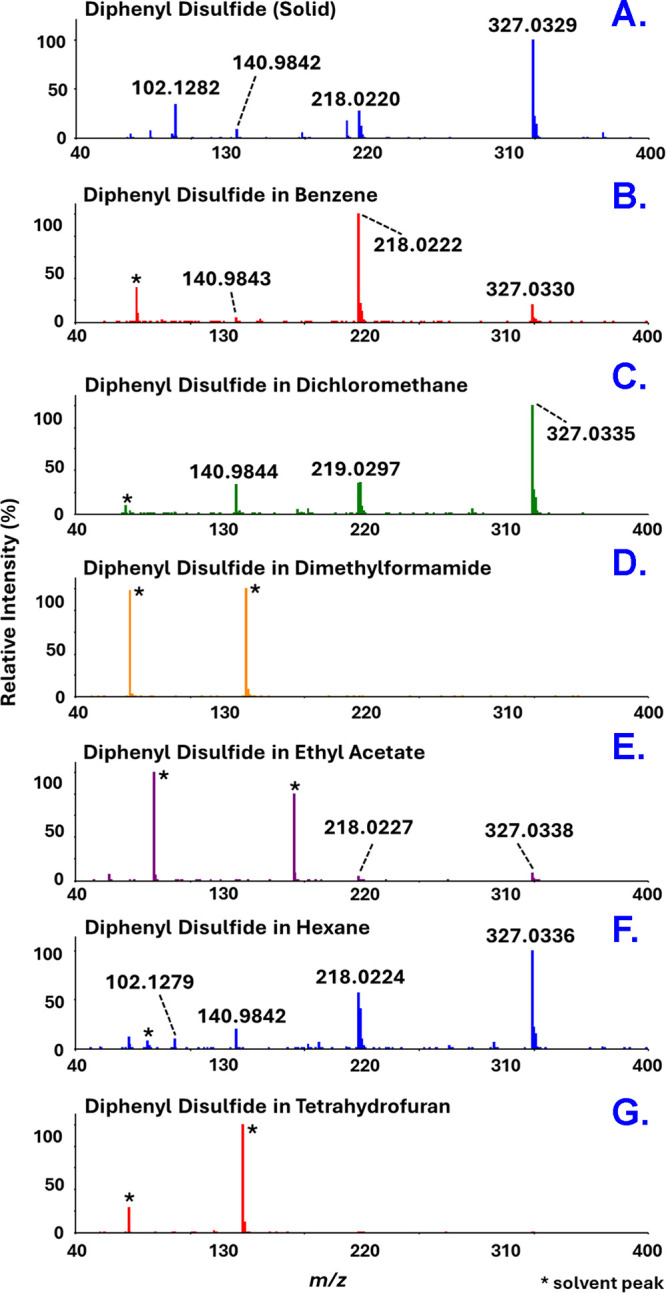
DART – high-resolution mass spectra of pure diphenyl disulfide (Panel A) and diphenyl disulfide dissolved in the indicated organic solvents (Panels B – G). The corresponding mass data tables appear in Document C of the Supporting Information.

The results show that although their relative intensities differ across the solutions analyzed, the peaks observed in the analysis of solutions in benzene, hexane, and DCM, correspond with those detected in the mass spectra of the corresponding pure neat/solid compounds. This outcome could be anticipated, given that the proton affinity (PA) of dimethyl disulfide (815.3 kJ/mol)^38^ (the only one available in the literature out of all the organic disulfides studied here), is greater than that of benzene (750.5 kJ/mol),^38^ hexane (672.8 kJ/mol),^39^ and DCM (628 ± 8 kJ/mol).^40^ As expected, peaks with *m*/*z* values consistent with the protonated masses of the solvent molecule were also detected (these are denoted by an asterisk (*)) in Figure 7 and Figures S10 – S17. On the other hand, the mass spectra of all of the disulfide molecules in DMF only showed two peaks: one at nominal *m*/*z* 74 and one at nominal *m*/*z* 147, which correspond to the protonated masses of DMF and its dimer, respectively. For none of the disulfides were peaks indicative of their presence observed in DMF. This outcome indicates that the PA of DMF (887.5 kJ/mol)^38^ is much greater than that of any of the disulfide compounds employed in this study, and therefore its ionization outcompeted that of the disulfides. Thus, in general, if the PA of the disulfide analyte is greater than that of the solvent, then *m*/*z* values consistent with those of both the disulfide and the solvent will be observed. However, if the PA of the solvent is greater than that of the disulfide, then only peaks representative of the solvent (i.e., the protonated precursor and protonated dimer) will be detected.

Results very similar to those observed for DMF were seen for solutions in THF and ethyl acetate (PAs of 822.1 and 835.7 kJ/mol, respectively),^38^ with one exception for the former and two for the latter. In most cases, analysis of THF solutions revealed a peak at nominal *m*/*z* 73 corresponding to the protonated mass of the solvent and a peak at nominal *m*/*z* 175 which is consistent with its protonated dimer. However, the mass spectrum collected during the analysis of dimesityl disulfide (C_18_H_22_S_2_, *calc*. 302.1163) in THF (Figure S10G) showed a peak at *m*/*z* 302.1161 that corresponded to the cation radical of the sulfide [C_18_H_22_S_2_]^+•^, and a peak at *m*/*z* 453.1742 consistent with an adduct composed of one dimesityl disulfide molecule plus a mesityl thiolate group [C_27_H_33_S_3_]^+^. The exceptions for ethyl acetate were observed for the diphenyl disulfide (Figure 7E) and diallyl disulfide (C_6_H_10_S_2_, *calc*. 146.0224, Figure S11E) solutions. All of the mass spectra of the ethyl acetate solutions showed solvent peaks at nominal *m*/*z* 89 and 177, consistent with the protonated masses of the solvent and its dimer. In addition to these two peaks, analysis of diallyl disulfide in ethyl acetate revealed the presence of *m*/*z* 131.0542, which corresponds to the protonated mass of diallyl sulfoxide [C_6_H_10_OS + H]^+^, and *m*/*z* 219.1055, which is consistent with the mass of diallyl disulfide plus an allyl thiolate group [C_9_H_15_S_3_]^+^. Among the solvent peaks, the mass spectrum of the diphenyl disulfide solution in ethyl acetate, as presented in Figure 7E, showed peaks at *m*/*z* 218.0227 and 327.0338, corresponding to the masses of its corresponding cation radical, and the diphenyl disulfide plus a phenyl thiolate group, respectively.

Overall, although the organosulfur molecules undergo fragmentation and recombination reactions even when in solution, the disulfide fragments formed in the He* DART gas stream do not appear to react with the solvent molecules, as no new unanticipated peaks were observed in the analyses. Furthermore, other solutions at lower concentrations (i.e., 10 and 100 ppm) were prepared for each organosulfur compound-solvent combination, and their analyses revealed the same results as described above.

### 3.7 Proposed Mechanism of Fragment and Adduct Formation

Based on the results of experimental measurements and theoretical calculations, a mechanistic framework is proposed to describe the initial dissociation pathways of disulfide compounds and the formation of their reaction products in the He* of the DART gas stream. This broadly applicable generalized mechanism which can be used to readily interpret the DART mass spectra of organic disulfides is presented in Figure 8. The neutral disulfide (R–S–S–R′) primarily undergoes S–S single-bond scission to form S-centered radicals such as R–S^•^ and R′–S^•^. This preference arises because the S–S bond dissociation energy is generally lower than those of the S–C and S–H bonds in disulfides. Further, R–S–S–R′ undergoes Penning ionization and proton-transfer processes, resulting in the formation of the radical cation [R–S–S–R′]^+•^ and the protonated ion [R–S–S–R′ + H]^+^. Following ionization, the radical cation [R–S–S–R′]^+•^ fragments via cleavage of the S−C bond under the present experimental conditions, producing carbon-centered and sulfur-centered intermediates, including R^•^, and R−S=S^+^ species (Figure 8). The formed reactive intermediates, such as R′−S^•^, R−S^•^, R^•^, and R−S=S^+^, subsequently participate in secondary reactions that give rise to products such as [R–S–R′]^+•^ and R–S^+^(S–R)–S–R′. In addition, reactions between [R–S–S–R′]^+•^ and OH radicals are observed under the analysis conditions, leading to the formation of oxygenated sulfur species, notably R–S^+^(OH)–S–R′.

**Figure 8:**
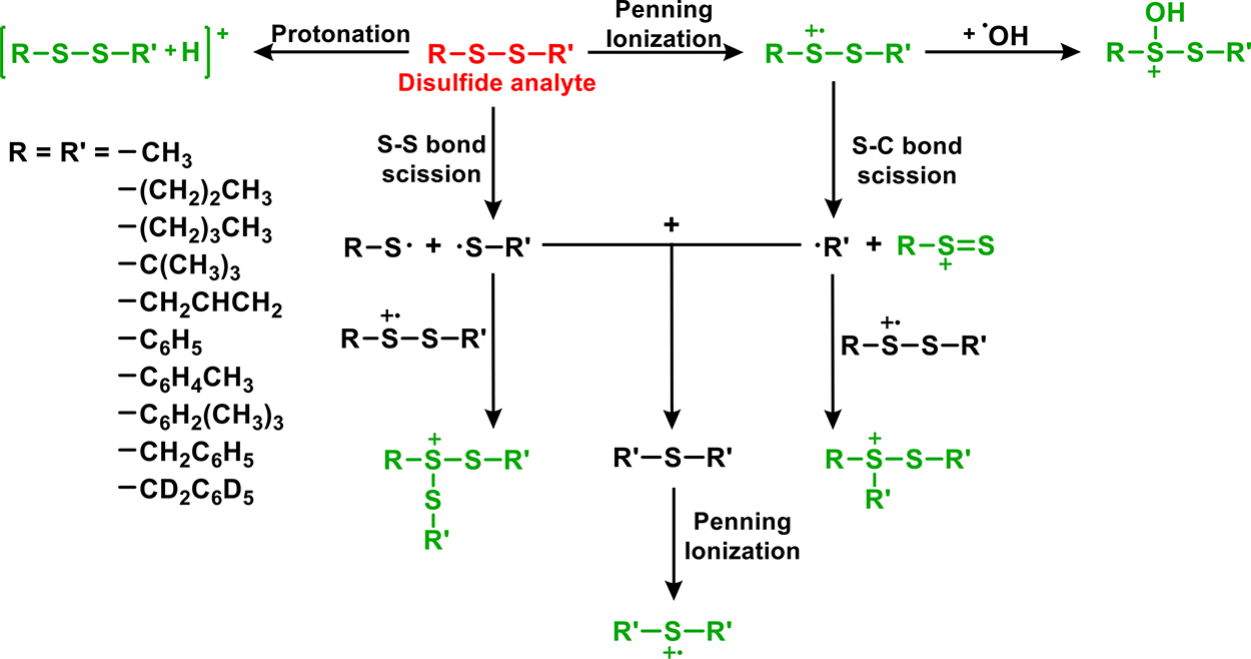
Proposed general mechanism for the formation of various ions observed in the DART – high-resolution mass spectra of disulfides. The ions highlighted in green are observed in the mass spectra obtained from the analysis of the pure disulfide compounds.

## 4 Conclusion

Analysis of pure organic disulfides by DART-HRMS revealed new entities that are formed on exposure of the analytes to the metastable helium of the DART gas stream, and these entities have been structurally characterized and probed by computational methods and tracer studies. The identities of the new organosulfur species were confirmed by DFT calculations. This study also demonstrated that the results hold true even in the presence of some solvents, provided that the disulfide molecules have proton affinity higher than that of the solvents in which they are dissolved.

## Supplementary Material







## Notes

Transition state geometries, PES profiles for the formation of the PhS^+^(OH)­SPh, PhSH••OSPh adduct, PhS^+^(SPh)­SPh, DART mass spectra of disulfide compounds (PDF)
Temperature study DART mass spectral tables of disulfide compounds analyzed by DART-HRMS at DART gas temperatures between 150 and 350 °C in increasing 25 °C increments (XLSX)
Solvent study DART mass spectral tables of disulfide compounds in organic solvents analyzed by DART-HRMS at DART gas temperature of 350 °C (XLSX)

## Notes

Present address: Rabi A. Musah, Louisiana State University, 232 Choppin Hall, Baton Rouge, Louisiana 70803, United States; (225) 578–8687, rmusah@lsu.edu
