# Supplementary material for: Experimental and Computational Investigation of Sulfur Chemistry in the DART-MS Gas Stream: Implications for the Interpretation of the Mass Spectra of Organic Disulfides
Source: J Am Soc Mass Spectrom. 2026 Apr 14;37(5):1268–80. doi: 10.1021/jasms.6c00046 (PMC13154360; doi:10.1021/jasms.6c00046)
Supplement: Supplementary file 1 [file js6c00046_si_001.pdf]

Supporting Information – A

Experimental and Computational Investigation of Sulfur Chemistry in the DART-MS Gas Stream: Implications for the Interpretation of the Mass Spectra of Organic Disulfides

Benedetta Garosi, Parandaman Arathala, and Rabi A. Musah*

Department of Chemistry, Louisiana State University, Baton Rouge, LA 70803, USA

**Corresponding author:* rmusah@lsu.edu *(R. A. Musah)*

Table of Contents

Figure S1. Transition state geometries for the formation of diphenyl disulfide-derived species 2

Figure S2. Scanned PES for the OH radical addition to the S-atom of the DPDS cation radical ...3

Figure S3. Scanned PES for the O-H bond scission of PhS(OH)SPh 4

Figure S4. Scanned PES for the addition of a PhS• to the S-atom of the DPDS cation radical........5

Figure S5. DART-HRMS mass spectrum of dibenzyl disulfide-d_{14}..6

Figure S6. DART-HRMS mass spectrum of dibenzyl disulfide.......................................7

Figure S7. DART-HRMS mass spectrum of diallyl disulfide 8

Figure S8. DART-HRMS mass spectrum of di-*n*-butyl disulfide 9

Figure S9. DART-HRMS mass spectrum of di-*p*-tolyl disulfide 10

Figure S10. DART-HRMS mass spectra of dimesityl disulfide in organic solvents 11

Figure S11. DART-HRMS mass spectra of diallyl disulfide in organic solvents 12

Figure S12. DART-HRMS mass spectra of dimethyl disulfide in organic solvents 13

Figure S13. DART-HRMS mass spectra of di-*n*-propyl disulfide in organic solvents 14

Figure S14. DART-HRMS mass spectra of di-*n*-butyl disulfide in organic solvents 15

Figure S15. DART-HRMS mass spectra of di-*tert*-butyl disulfide in organic solvents 16

Figure S16. DART-HRMS mass spectra of di-*p*-tolyl disulfide in organic solvents 17

Figure S17. DART-HRMS mass spectra of dibenzyl disulfide in organic solvents 18

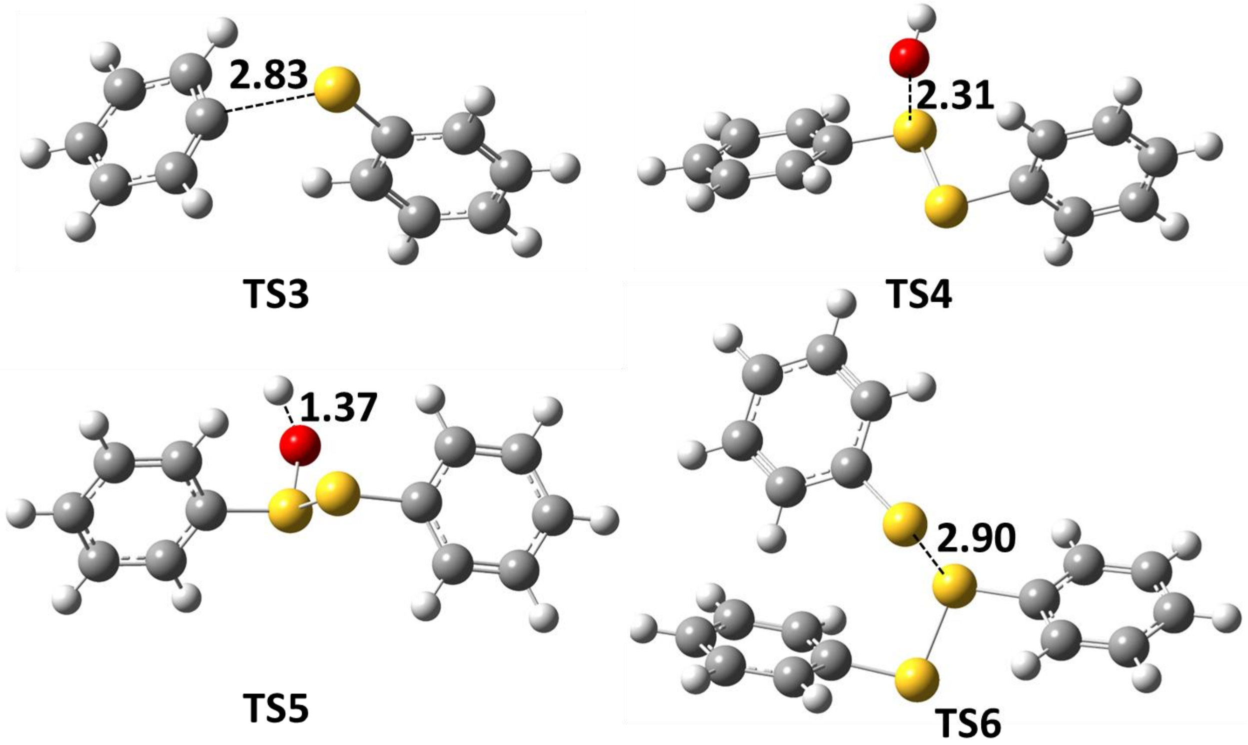

Figure S1. Transition state geometries for the formation of diphenyl sulfide cation radical, $PhS^{+}(OH)SPh$, $[PhS(=O)SPh + H]^{+}$, Ph-S^{+}(S-Ph)-S-Ph optimized at the M06-2X/aug-cc-pV(T+d)Z level of theory. The carbon, hydrogen, oxygen and sulfur atoms are shown in black, white, red and yellow colors, respectively.

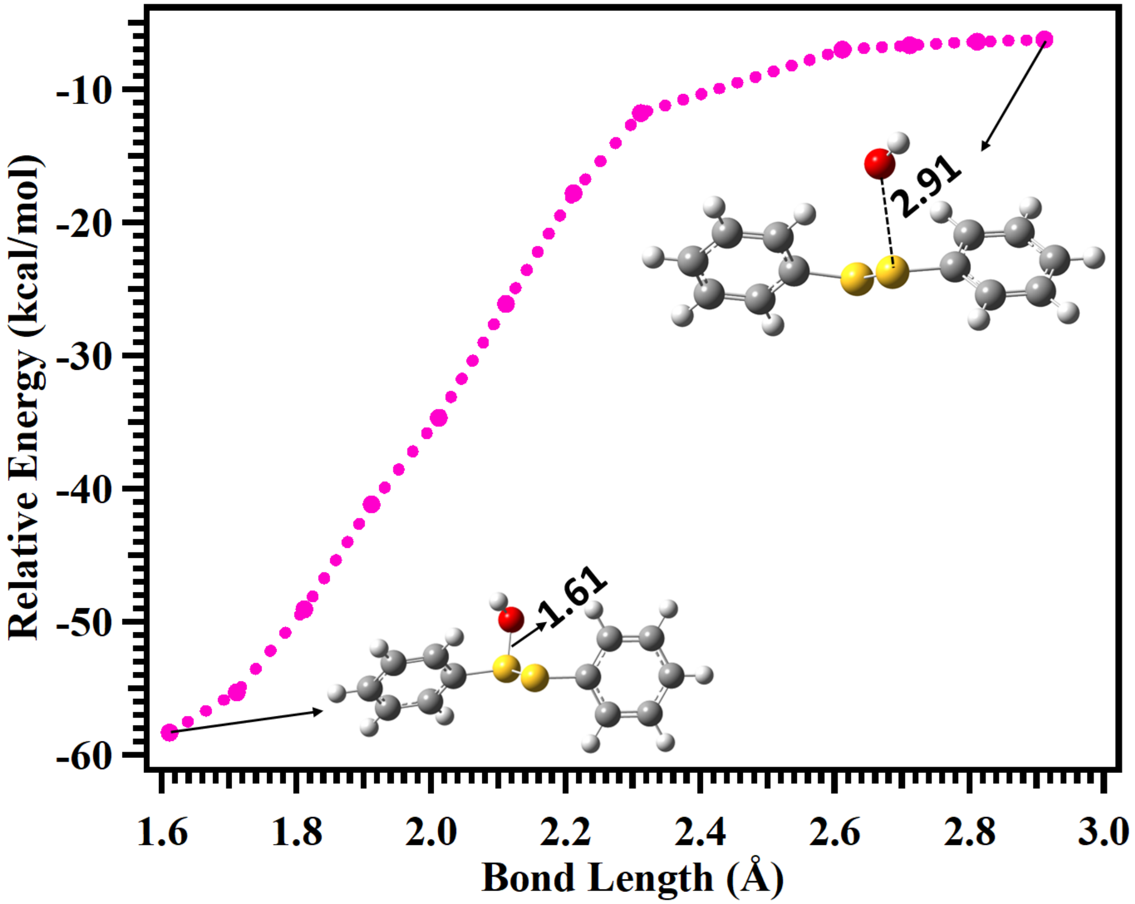

Figure S2. Scanned potential energy surface for OH radical addition to the S-atom of the DPDS cation radical leading to the formation of $PhS^{+}(OH)SPh$ at M06-2X/aug-cc-pV(T+d)Z level of theory. The relative energies were calculated with respect to the energy of $[PhSSPh]^{+\bullet}$ + $^{\bullet}OH$ as reactants.

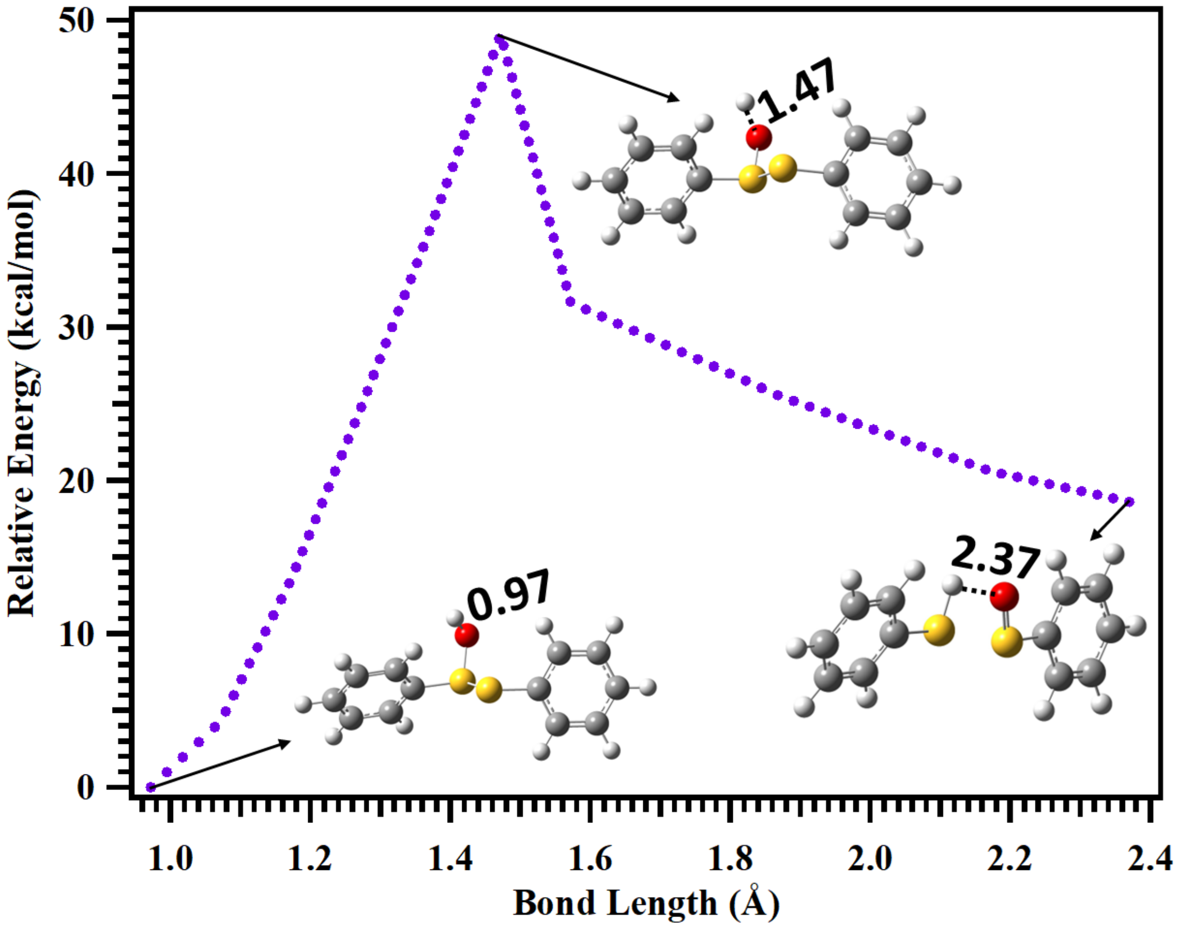

Figure S3. Scanned potential energy surface for the O-H bond scission of $PhS^{+}(OH)SPh$ leading to formation of PhSH••OSPh at the M06-2X/aug-cc-pV(T+d)Z level of theory. The relative energies were calculated with respect to the energy of $PhS^{+}(OH)SPh$.

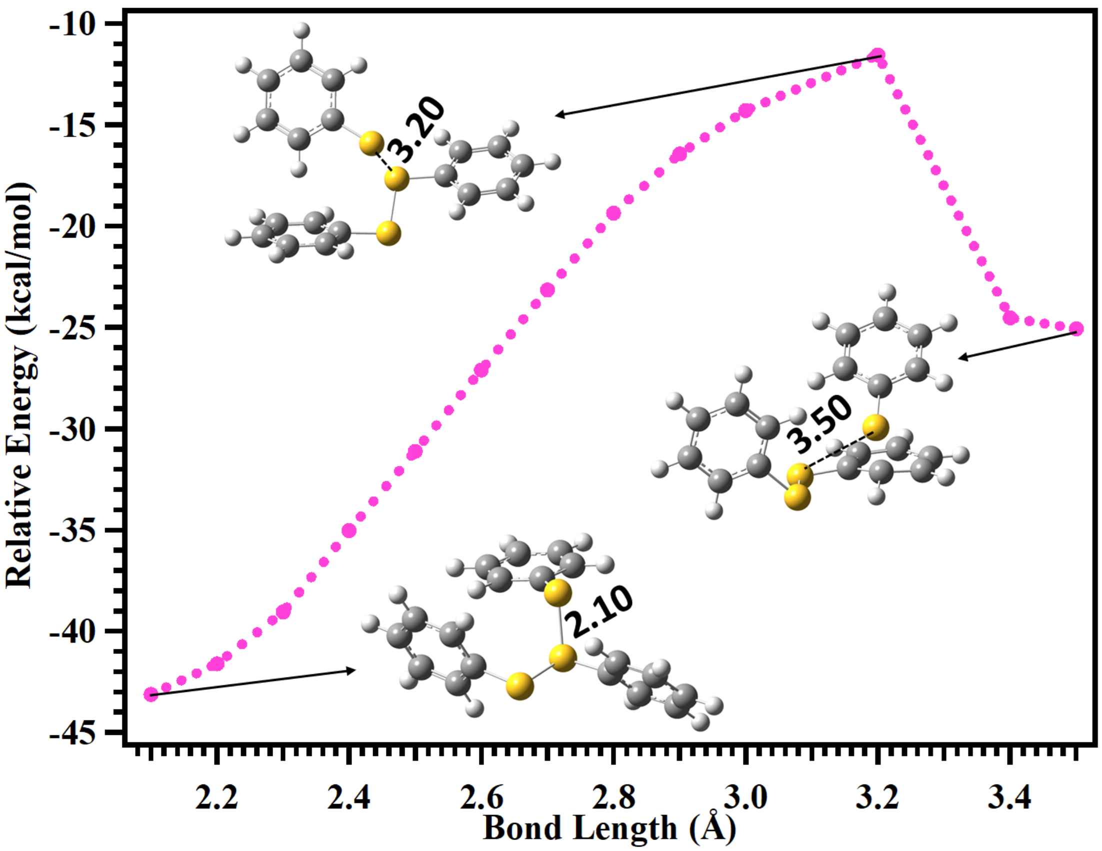

Figure S4. Scanned potential energy surface for the addition of a phenyl thiyl radical to the sulfur atom of the DPDS cation radical leading to formation of $PhS^{+}(SPh)SPh$ at the M06-2X/aug-cc-pV(T+d)Z level of theory. The relative energies were calculated with respect to the energy of $PhSSPh^{+\bullet}$ + $PhS^{\bullet}$ as reactants.

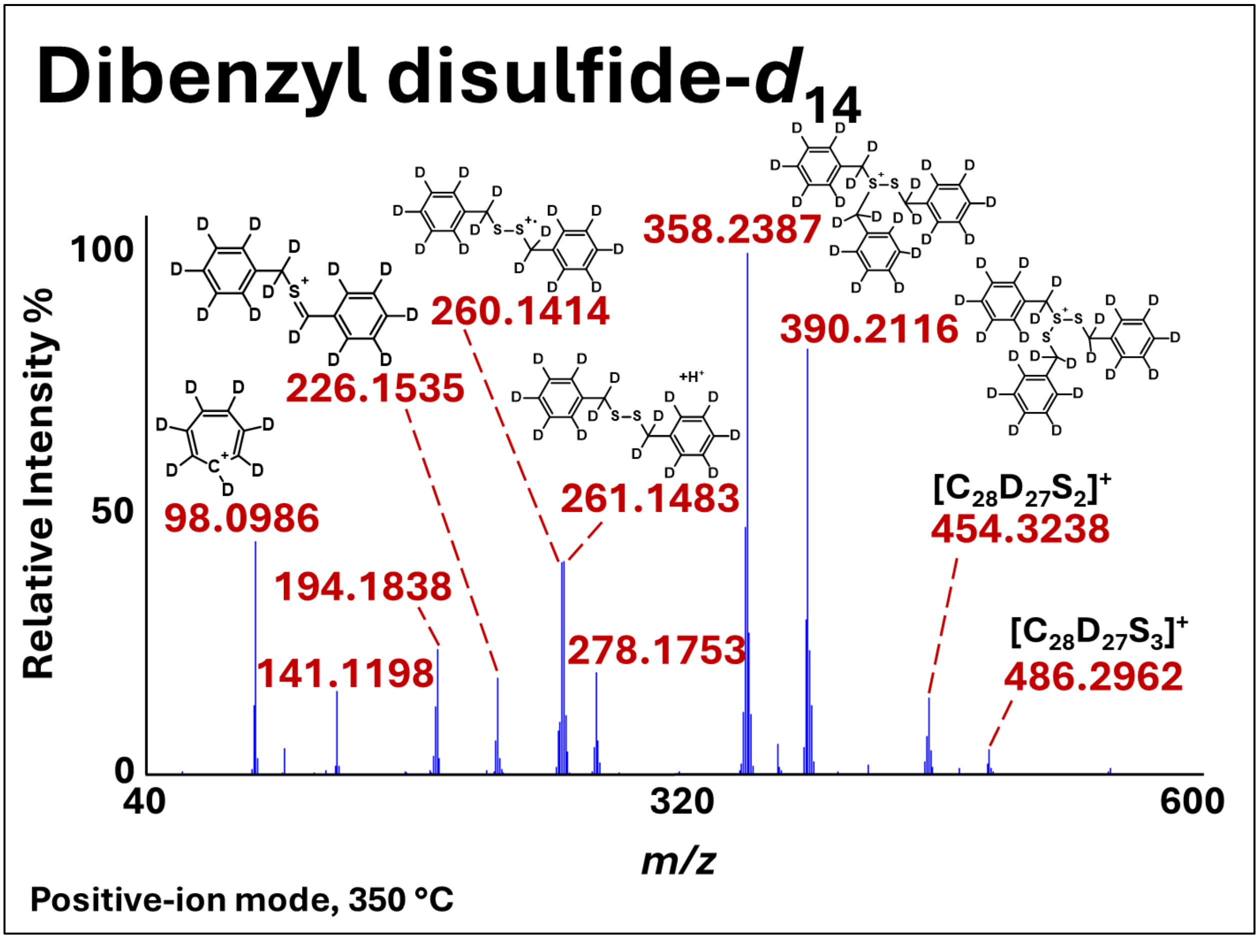

Figure S5. DART mass spectrum of dibenzyl disulfide-d_{14} analyzed under soft ionization conditions in positive-ion mode at a He* gas temperature of 350 °C. Each identified peak is labeled with its respective observed *m/z* value and proposed structure.

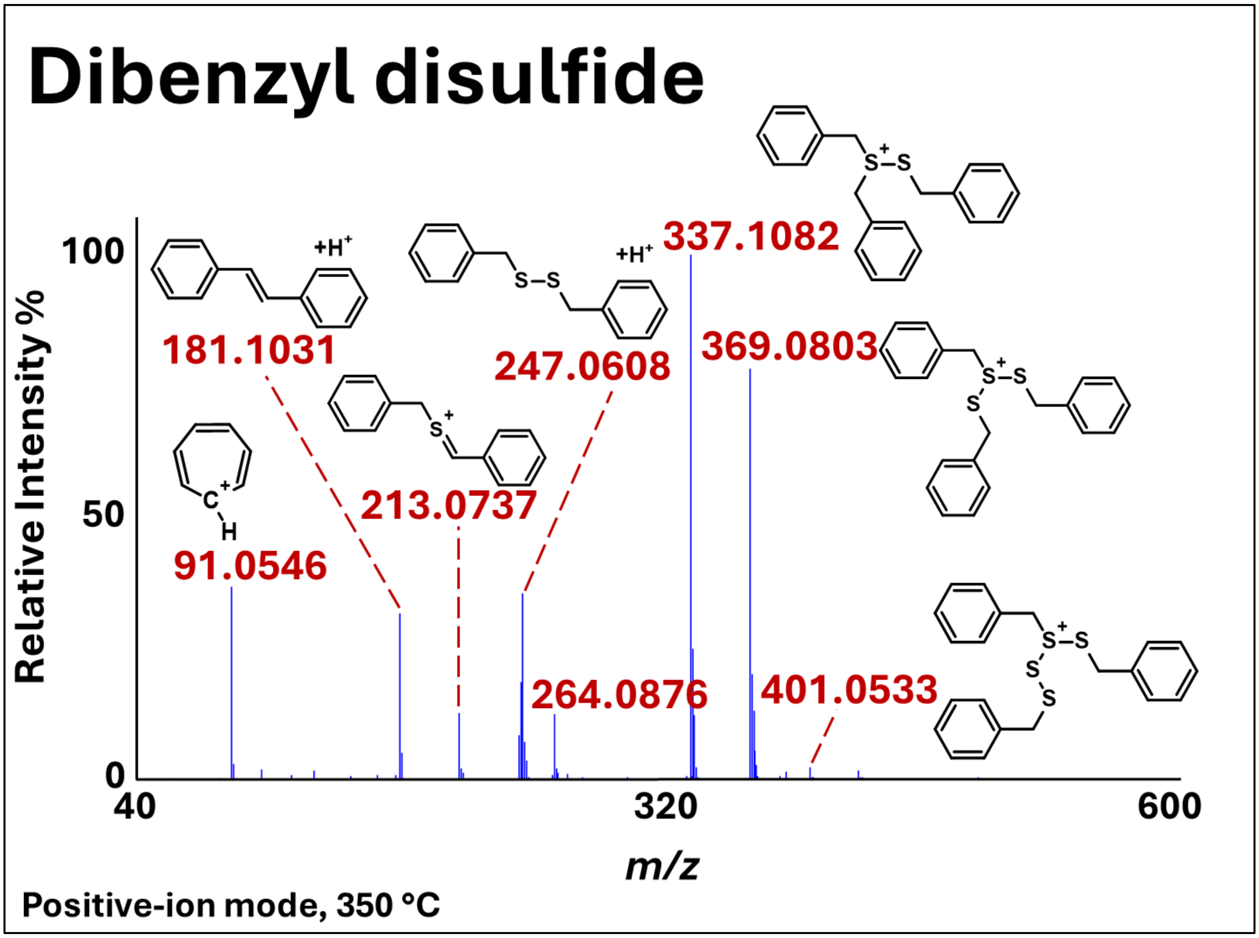

Figure S6. DART mass spectrum of dibenzyl disulfide analyzed under soft ionization conditions in positive-ion mode at a He* gas temperature of 350 °C. Each identified peak is labeled with its respective observed *m/z* value and proposed structure.

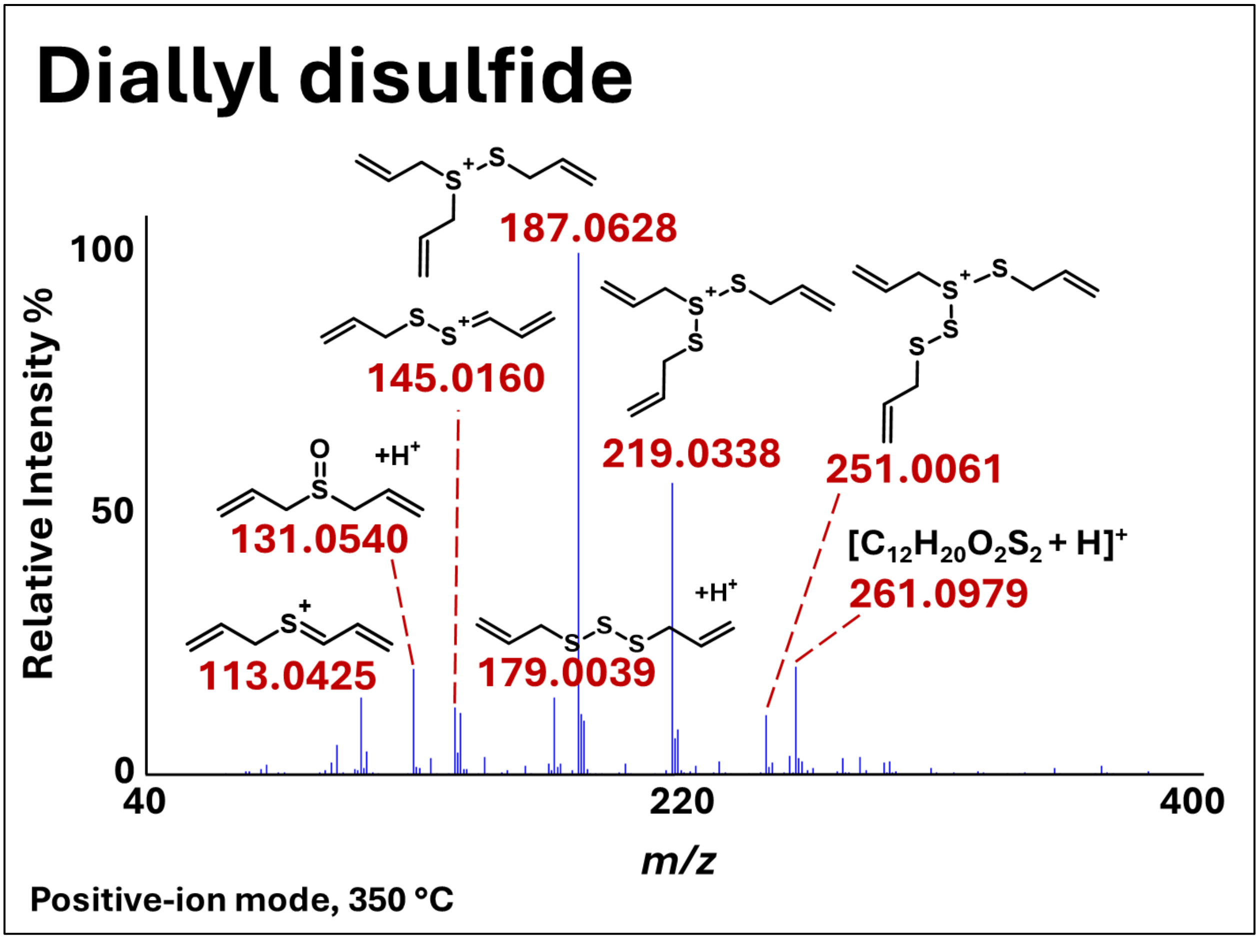

Figure S7. DART mass spectrum of diallyl disulfide analyzed under soft ionization conditions in positive-ion mode at a He* gas temperature of 350 °C. Each identified peak is labeled with its respective observed *m/z* value and proposed structure.

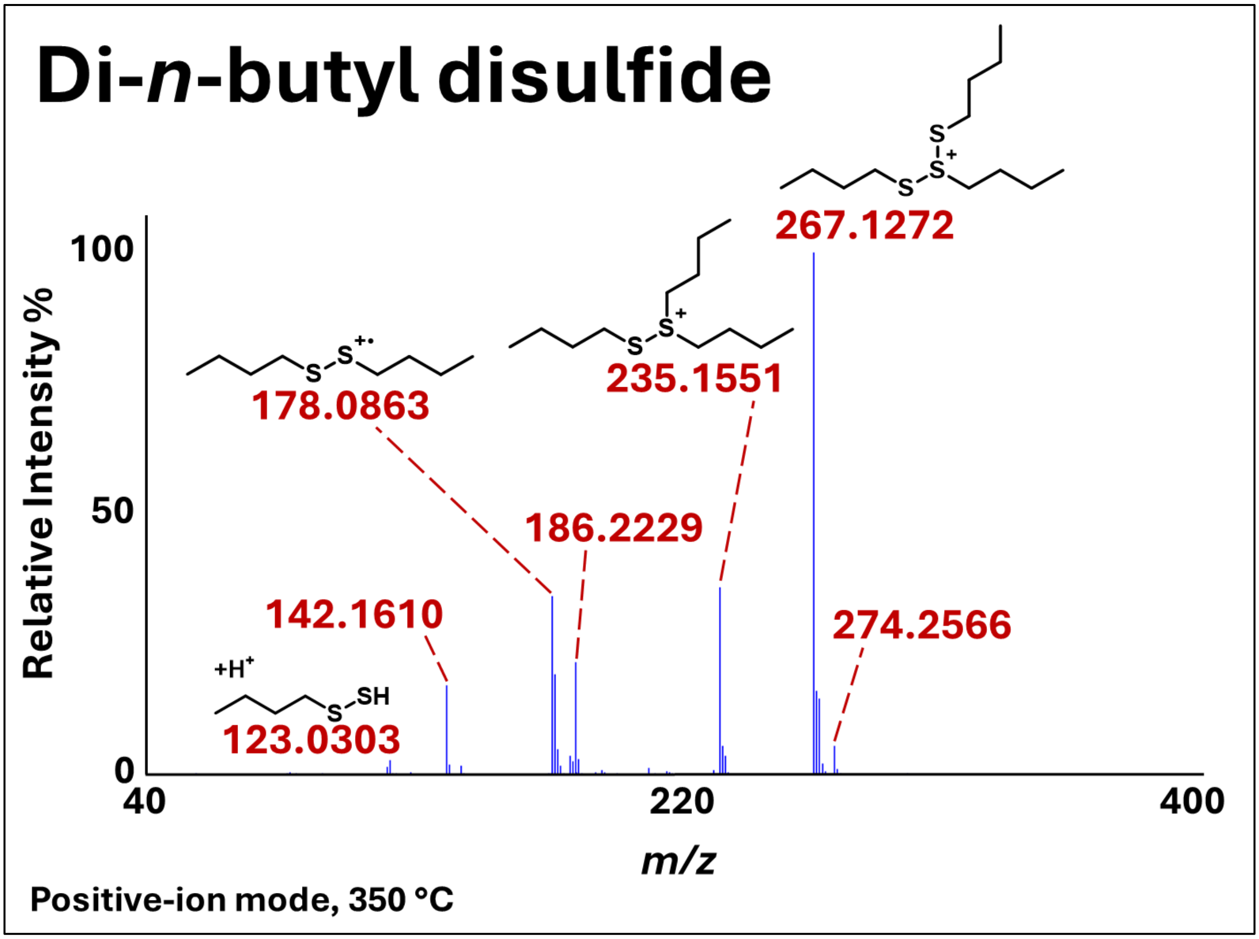

Figure S8. DART mass spectrum of di-*n*-butyl disulfide analyzed under soft ionization conditions in positive-ion mode at a He* gas temperature of 350 °C. Each identified peak is labeled with its respective observed *m/z* value and proposed structure.

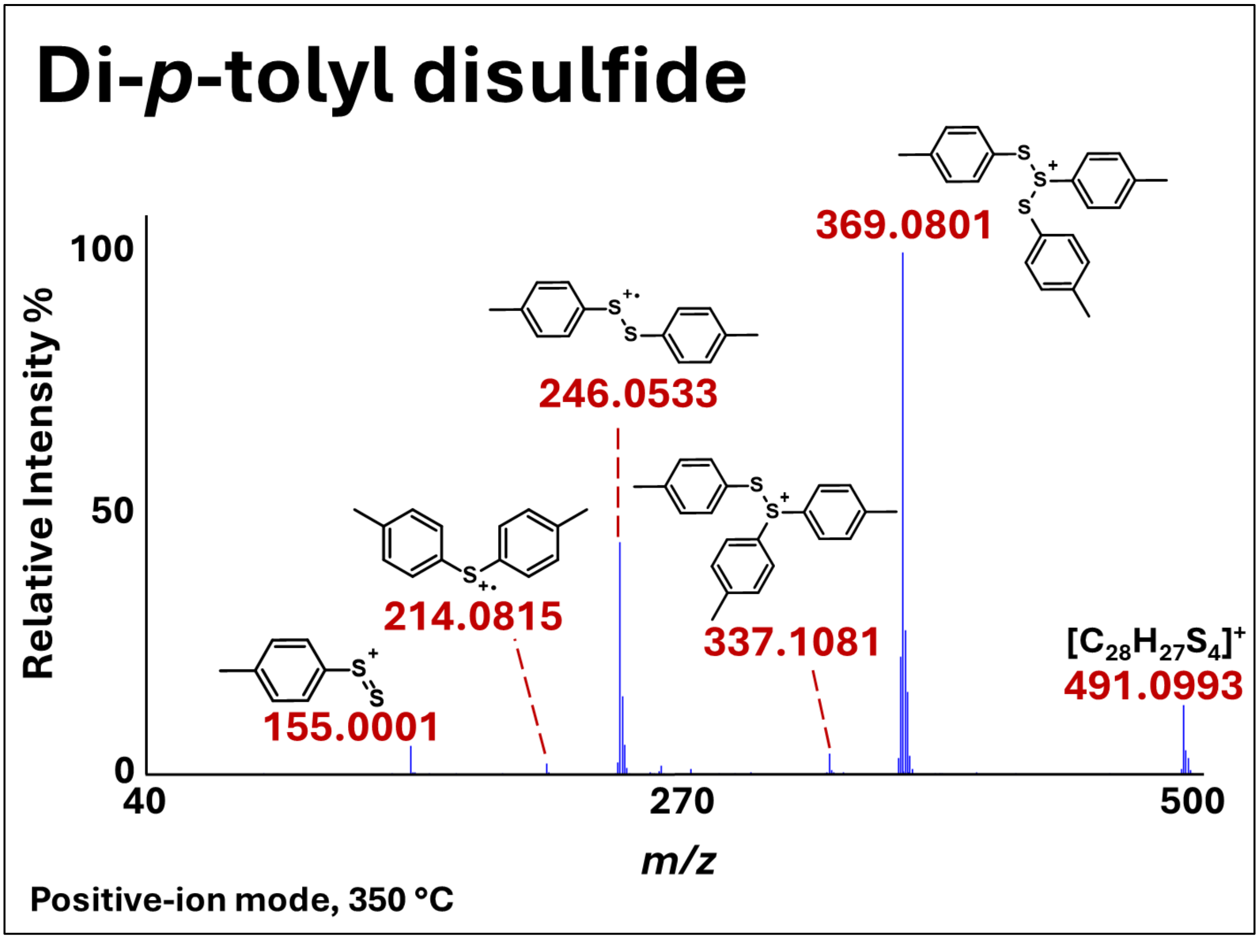

Figure S9. DART mass spectrum of di-*p*-tolyl disulfide analyzed under soft ionization conditions in positive-ion mode at a He* gas temperature of 350 °C. Each identified peak is labeled with its respective observed *m/z* value and proposed structure.

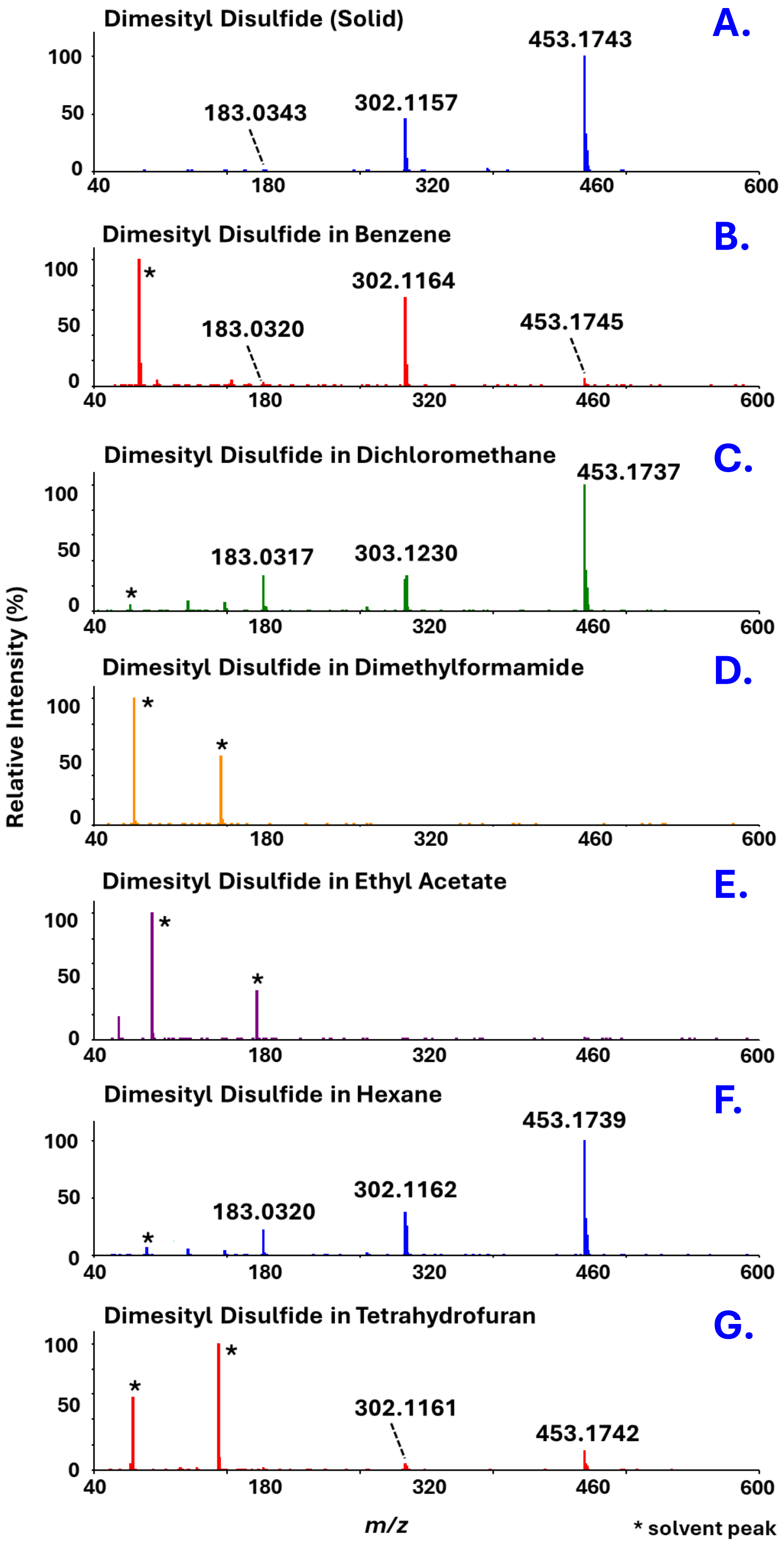

Figure S10. DART – high-resolution mass spectra of pure dimesityl disulfide (Panel A) and dimesityl disulfide dissolved in the indicated organic solvents (Panels B – G).

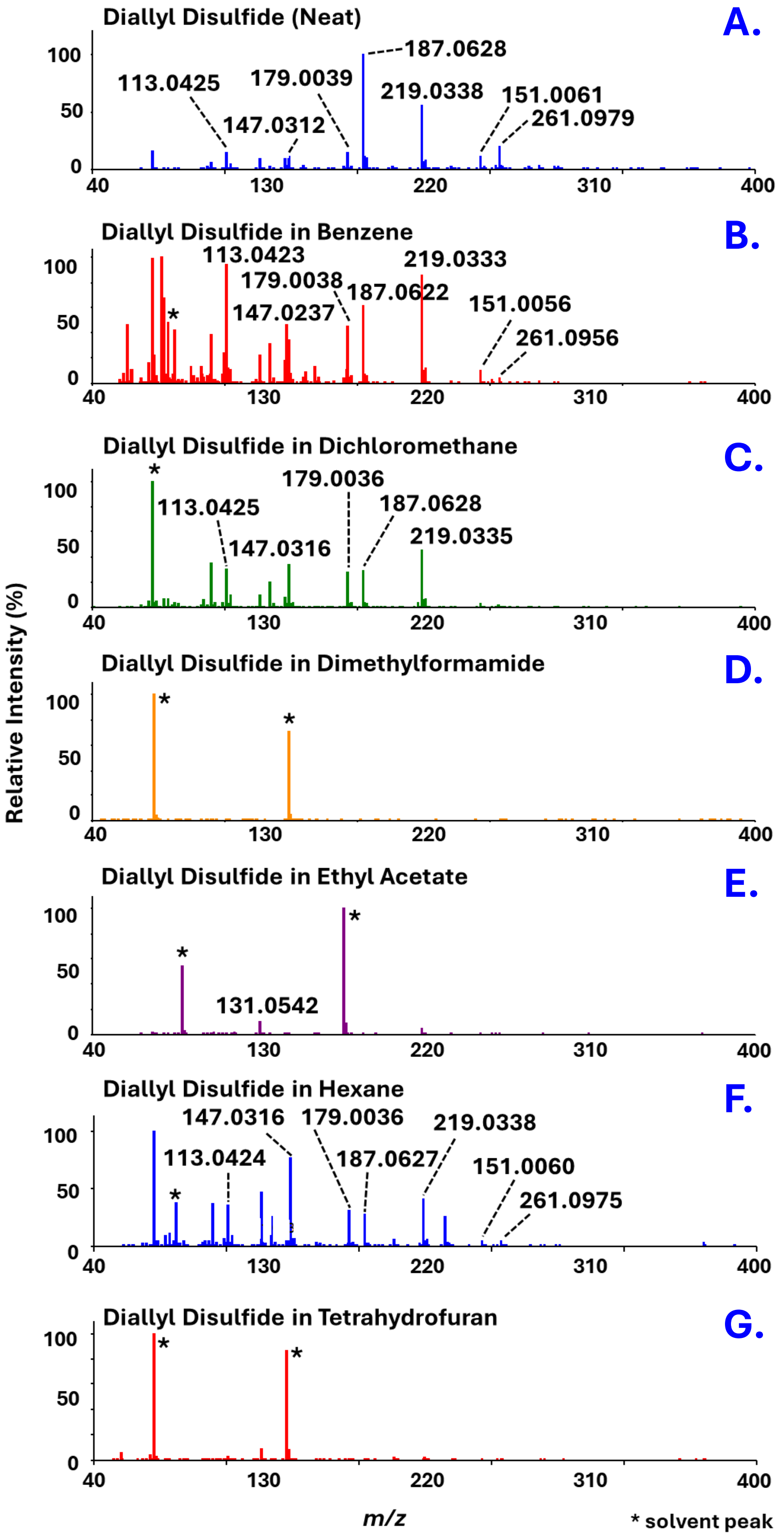

Figure S11. DART – high-resolution mass spectra of pure diallyl disulfide (Panel A) and diallyl disulfide dissolved in the indicated organic solvents (Panels B – G).

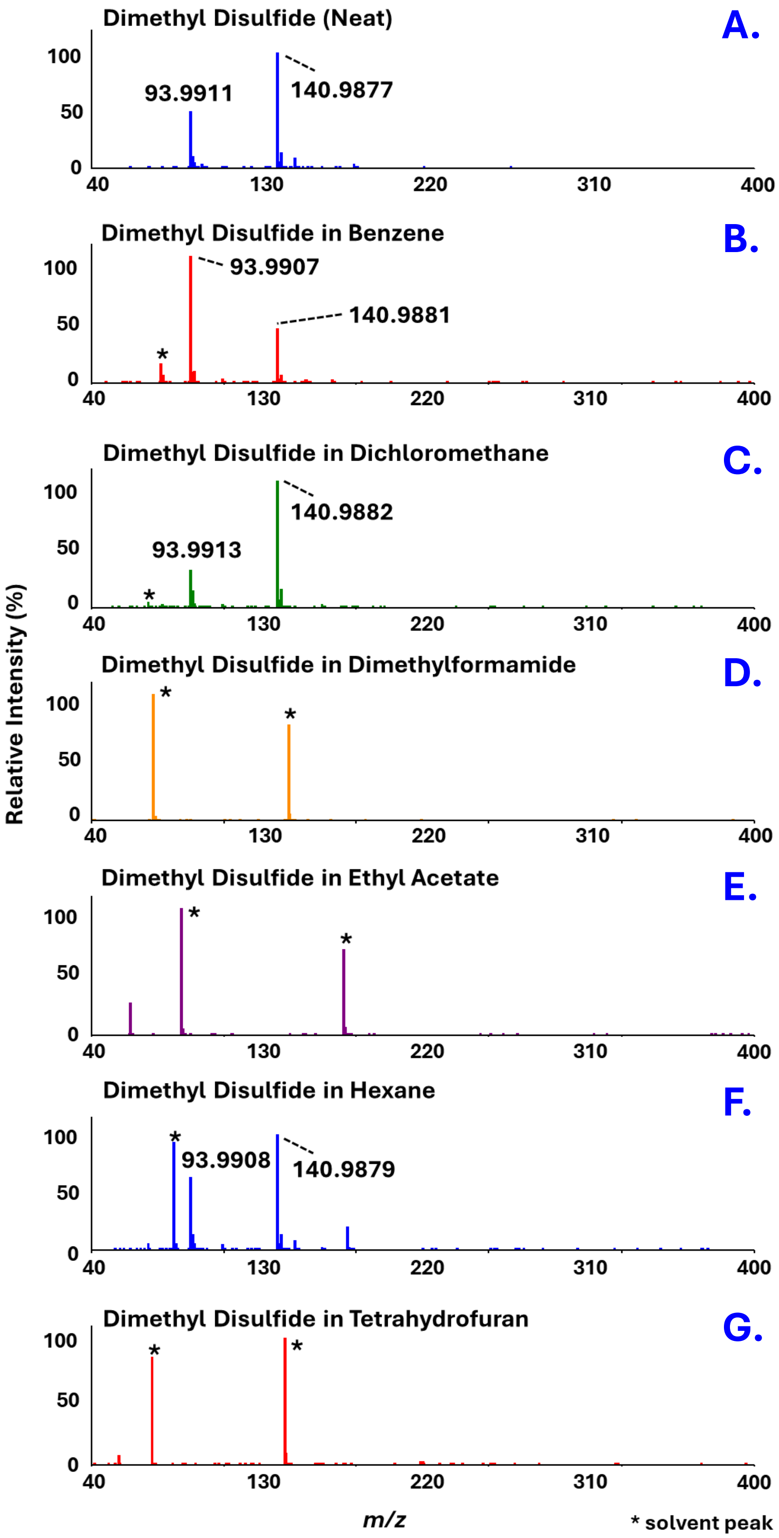

Figure S12. DART – high-resolution mass spectra of pure dimethyl disulfide (Panel A) and dimethyl disulfide dissolved in the indicated organic solvents (Panels B – G).

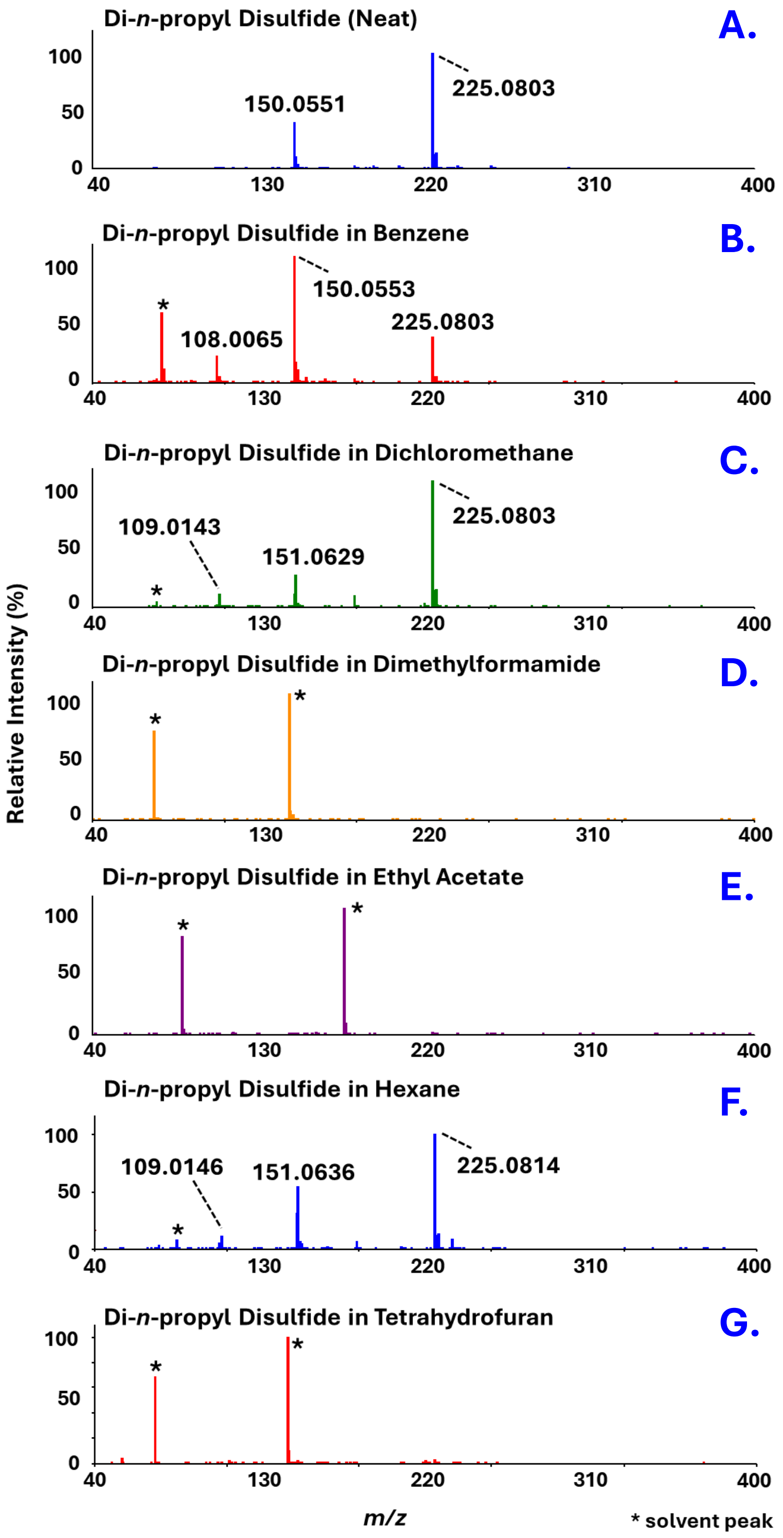

Figure S13. DART – high-resolution mass spectra of pure di-*n*-propyl disulfide (Panel A) and di-*n*-propyl disulfide dissolved in the indicated organic solvents (Panels B – G).

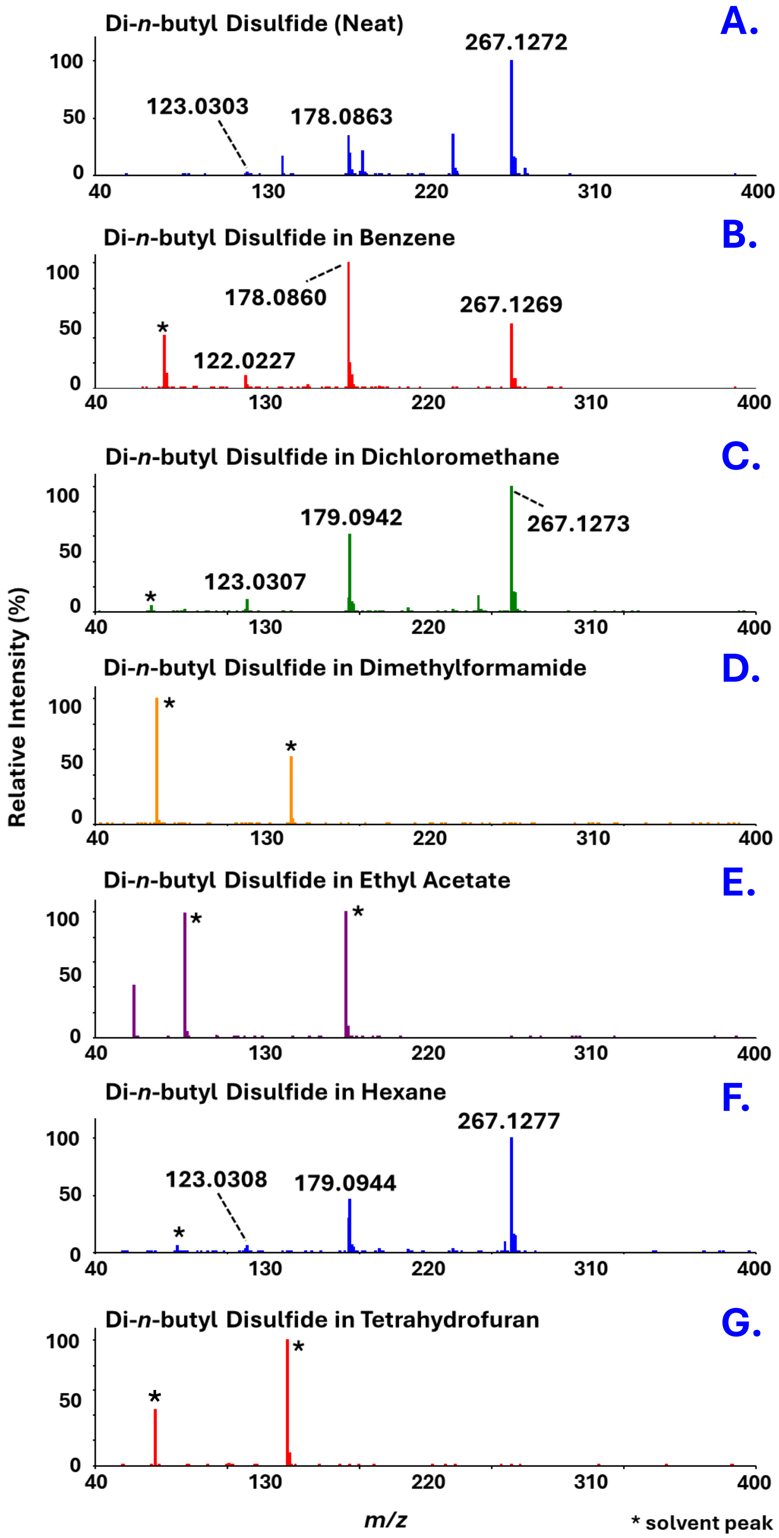

Figure S14. DART – high-resolution mass spectra of pure di-*n*-butyl disulfide (Panel A) and di-*n*-butyl disulfide dissolved in the indicated organic solvents (Panels B – G).

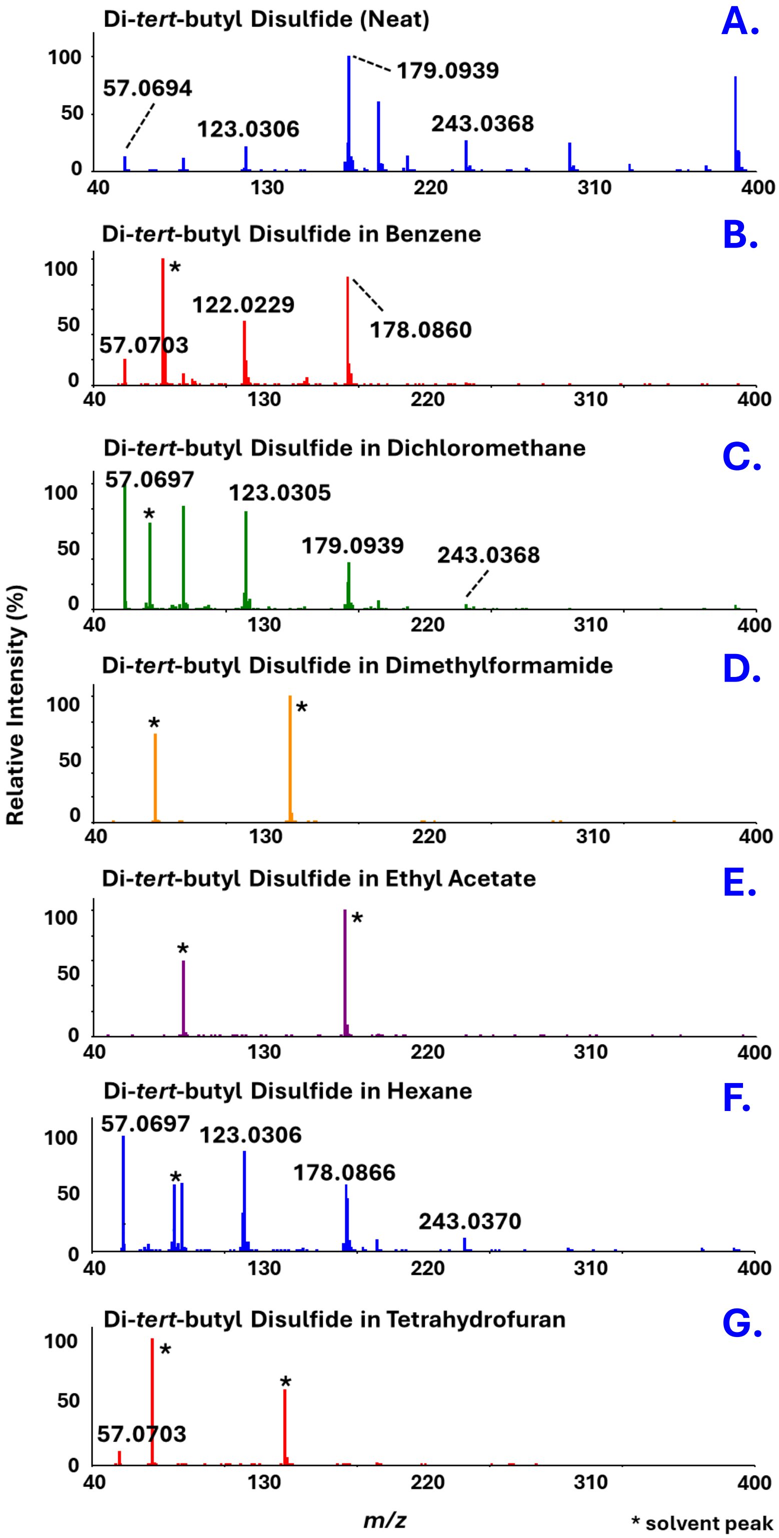

Figure S15. DART – high-resolution mass spectra of pure di-*tert*-butyl disulfide (Panel A) and di-*tert*-butyl disulfide dissolved in the indicated organic solvents (Panels B – G).

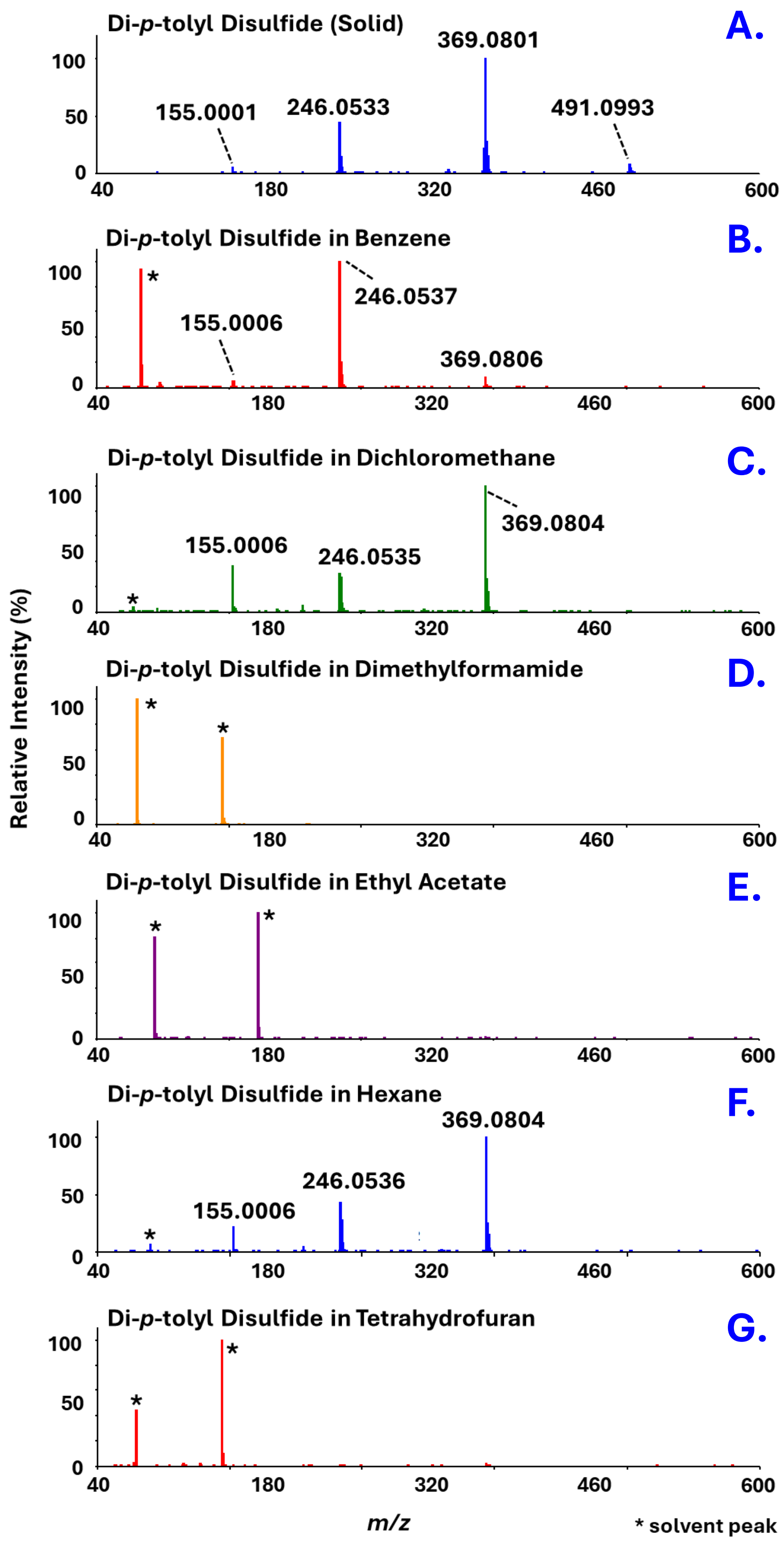

Figure S16. DART – high-resolution mass spectra of pure di-*p*-tolyl disulfide (Panel A) and di-*p*-tolyl disulfide dissolved in the indicated organic solvents (Panels B – G).

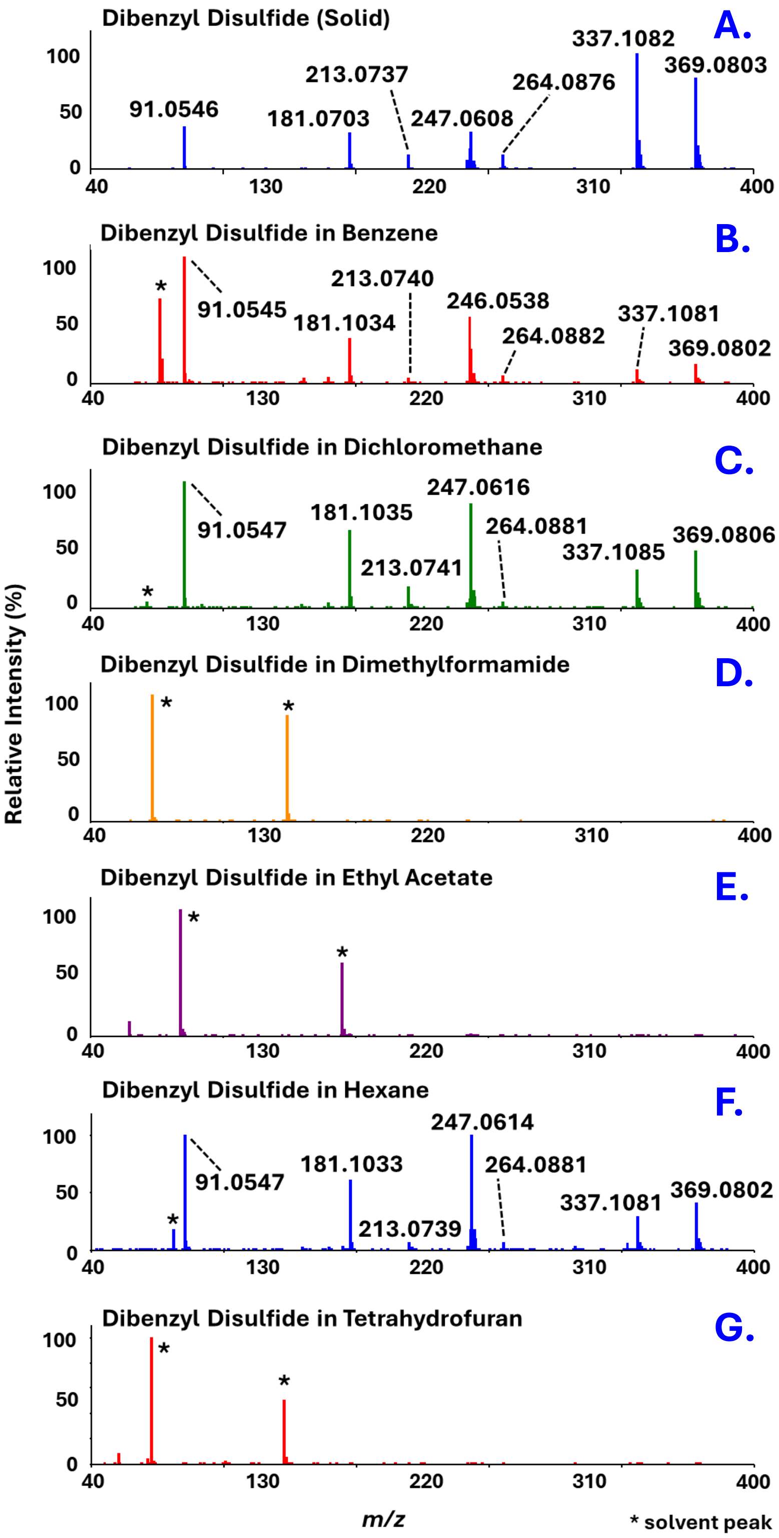

Figure S27. DART – high-resolution mass spectra of pure dibenzyl disulfide (Panel A) and dibenzyl disulfide dissolved in the indicated organic solvents (Panels B – G).
